# Metabolic dysregulation and biological age acceleration in Hashimoto’s thyroiditis: a cross-sectional study based on clinical biomarker aging indices and metabolomics

**DOI:** 10.3389/fendo.2026.1874574

**Published:** 2026-06-26

**Authors:** Xinyu Zhao, QunHao Li, Tao Luo, Wenxuan Fang, Qian Liu, Hao Li, Jie Su, Xiao Jiang, Jialan Yu

**Affiliations:** 1 Department of Cadre Ward I, PLA No. 967 Hospital of the Joint Support Force, Dalian, Liaoning, China; 2 Key Laboratory of Endocrine Glucose and Lipids Metabolism and Brain Aging, Ministry of Education; Department of Endocrinology, Shandong Provincial Hospital Affiliated to Shandong First Medical University, Jinan, Shandong, China; 3 Department of Gastroenterology, Huaihai Hospital Affiliated to Xuzhou Medical University, Xuzhou, Jiangsu, China; 4 Graduate School Department, Dalian Medical University, Dalian, Liaoning, China

**Keywords:** biological age, citric acid, Hashimoto’s thyroiditis, metabolic age, metabolomics

## Abstract

**Background:**

Hashimoto’s thyroiditis (HT) is a common autoimmune thyroid disease characterized by thyroid autoantibody positivity, chronic lymphocytic inflammation, and varying degrees of thyroid dysfunction. Whether HT is associated with quantifiable biological age acceleration and metabolic age-related remodeling remains insufficiently examined.

**Methods:**

This cross-sectional study included two clinical discovery cohorts, an NHANES 2007–2012 validation cohort, and a metabolomics cohort. Klemera-Doubal method biological age (KDM biological age), Phenotypic Age (PhenoAge), and corresponding age acceleration metrics were calculated from clinical biomarkers, and the proportions of participants with age acceleration were compared. In the metabolomics cohort, a random forest metabolic age model was trained in healthy controls and internally evaluated by five-fold cross-validation. Metabolic age acceleration (MAA) was defined as predicted metabolic age minus chronological age. Metabolites associated with age, FT3, FT4, and TSH, together with differential metabolites in euthyroid HT (EHT) and thyroid dysfunction HT (DHT) relative to controls, were integrated to screen candidate metabolites and construct exploratory prediction models.

**Results:**

In the two discovery cohorts, the HT group showed higher KDM biological age and/or PhenoAge indices and higher proportions of age acceleration than healthy controls. In the NHANES validation cohort, after adjustment for age, sex, socioeconomic factors, and lifestyle factors, overall HT was associated with higher KDM biological age (beta = 3.16 years, 95% CI 1.77-4.56) and PhenoAge (beta = 1.54 years, 95% CI 1.05-2.03). Stage-specific analyses suggested relatively stable associations for euthyroid HT and subclinical hypothyroid HT, whereas results for overt hypothyroid HT should be interpreted cautiously because of the small sample size and wide confidence intervals. In the random forest metabolic age model, the mean test-set performance across five-fold cross-validation was RMSE 8.75 years, MAE 7.11 years, R 0.634, and R2 0.427. MAA differed among CON, EHT, and DHT groups (Kruskal-Wallis P = 6.6 x 10^-5), and both EHT and DHT had higher MAA than CON after adjustment for age and sex. Integrated analysis identified 18 candidate metabolites; the HT classification model based on these metabolites had an AUC of 0.980, and predicted values from the FT3, FT4, and TSH models were significantly correlated with the measured values. Considering clinical relevance, between-group expression trends, and model-intersection evidence, citric acid, LPC 20:0 sn-1, and SM 34:2 were prioritized as core candidate metabolites.

**Conclusions:**

In the cross-sectional cohorts included in this study, HT was associated with higher biological age and metabolic age acceleration. Metabolomics results suggest that citric acid-related energy metabolism and lipid molecules such as LPC and SM may reflect HT- and thyroid-function-related metabolic features.

## 1 Introduction

Aging is not merely the accumulation of chronological time; it is a complex biological process involving genomic stability, mitochondrial function, metabolic homeostasis, cellular senescence, and remodeling of immune-inflammatory networks. In recent years, aging research has gradually shifted from stratification by chronological age to characterization by biological age, because biological age more accurately reflects interindividual heterogeneity in health deterioration and chronic disease risk than chronological age (1, 2). Within this framework, chronic low-grade inflammation (inflammaging) is considered an important shared mechanism driving multiple age-related diseases (3, 4).

Hashimoto’s thyroiditis (HT) is the most common autoimmune thyroid disease and one of the major causes of hypothyroidism in iodine-sufficient regions (5, 6). Its pathological basis includes persistent lymphocytic infiltration, destruction of thyroid follicles, thyroid autoantibody positivity, and progressive thyroid dysfunction (5). The thyroid axis itself is closely linked to aging; with increasing age, thyroid hormone secretion, metabolism, and target-organ sensitivity may change, while thyroid dysfunction may in turn influence metabolism, cardiovascular health, muscle function, cognition, and other aging-related systems (6, 7). Therefore, whether HT forms a quantifiable pro-aging phenotype warrants systematic evaluation.

Klemera-Doubal biological age (KDM biological age) and Phenotypic Age (PhenoAge) are widely used biological age quantification tools. The former estimates biological age based on the joint associations between multiple clinical biomarkers and chronological age, whereas the latter emphasizes physiological dysregulation related to morbidity and mortality risk (8, 9). However, evidence on the association between HT and biological age remains limited, particularly studies combining discovery cohorts with external validation.

Metabolomics provides a new entry point for understanding systemic changes in HT. Previous studies suggest that HT and related thyroid dysfunction may be accompanied by marked remodeling of lipid metabolism, amino acid metabolism, and energy metabolism, particularly abnormalities in sphingomyelin (SM), lysophosphatidylcholine (LPC), phosphatidylcholine (PC), and some tricarboxylic acid cycle-related metabolites (10–12). Citric acid is not only a core intermediate of the TCA cycle but also participates in inflammatory immune reprogramming, ATP-citrate lyase-related inflammatory responses, and cellular senescence-associated metabolic alterations, making it a potential signaling hub linking energy metabolism and inflammatory aging (13–16).

Based on these considerations, this study systematically evaluated the pro-aging features of HT at three levels. First, the association between HT and aging phenotypes was assessed from the perspective of biological age in discovery cohorts and the NHANES validation cohort. Second, a metabolic age model was constructed to examine metabolic age changes across functional states of HT. Third, metabolomics was used to identify core metabolites simultaneously related to aging, HT diagnosis, and disease progression (increased TSH and decreased FT3/FT4), and their potential bridging roles were discussed.

## 2 Materials and methods

### 2.1 Study design and data sources

This study included four cohorts: discovery cohort 1, discovery cohort 2, the NHANES validation cohort, and the metabolomics cohort. According to the current analysis tables, discovery cohort 1 included 249 healthy controls and 159 patients with HT; discovery cohort 2 included 27 healthy controls and 31 HT patients; the NHANES validation cohort included 2,483 healthy controls and 705 HT patients; and the metabolomics cohort included 77 healthy controls (CON), 48 patients with euthyroid HT (EHT), and 36 patients with subclinical hypothyroid HT (DHT). Additional cohort descriptions are provided in **Supplementary Materials 1**.

Discovery cohort 1 was derived from the health examination population at Shandong Provincial Third Hospital from 2023 to 2026, and discovery cohort 2 from the health examination population at Dalian 967 Hospital from 2024 to 2026. Both cohorts included participants with complete thyroid function data and clinical indicators required for biological age calculation. HT was diagnosed according to: (1) thyroid ultrasonography showing diffuse thyroid enlargement, a rough surface, or nodular changes; and (2) positivity for anti-thyroglobulin antibody (TgAb) and/or thyroid peroxidase antibody (TPOAb).

In the metabolomics cohort, inclusion criteria for EHT were: (1) thyroid ultrasound showing diffuse thyroid enlargement, a rough surface, or nodular changes; (2) TgAb and/or TPOAb positivity; (3) serum free triiodothyronine (FT3), free thyroxine (FT4), and thyroid-stimulating hormone (TSH) within reference ranges; and (4) no related drug treatment. Inclusion criteria for DHT were: (1) thyroid ultrasound showing diffuse thyroid enlargement, a rough surface, or nodular changes; (2) positivity for both TgAb and TPOAb; (3) elevated serum TSH, with FT3 and FT4 within the normal range or decreased; and (4) no related drug treatment.

Exclusion criteria for all cohorts were: (1) other autoimmune diseases, such as diabetes, systemic lupus erythematosus, or inflammatory bowel disease; (2) acute or chronic infectious diseases, such as acute or chronic hepatitis or pneumonia; (3) current use of non-steroidal anti-inflammatory drugs or glucocorticoids; (4) malignant tumors or immunodeficiency; and (5) pregnancy or lactation.

The NHANES validation cohort used participants from NHANES 2007–2012 with complete thyroid function data and clinical indicators for biological age construction. Minors and pregnant women were excluded; the detailed inclusion and exclusion process is shown in **Supplementary Materials 2**. Covariates with missingness below 10% in NHANES, including smoking, alcohol intake, and physical activity, were imputed using K-nearest neighbors (KNN). Diagnostic criteria in NHANES were as follows: HT with euthyroidism: (1) TgAb and/or TPOAb positivity; and (2) normal serum TSH, with FT3 and FT4 within the normal range or decreased. HT with subclinical hypothyroidism: (1) TgAb and/or TPOAb positivity; and (2) elevated serum TSH with normal FT3 and FT4. HT with overt hypothyroidism: (1) TgAb and/or TPOAb positivity; and (2) elevated serum TSH with decreased FT3 and FT4. In the initial analysis, 480 individuals with Hashimoto’s thyroiditis (HT) and euthyroidism, 56 with HT and subclinical hypothyroidism, and 45 with HT and overt hypothyroidism were included.

To assess the potential impact of thyroid-related medications on the findings, we performed a sensitivity analysis in the NHANES cohort by excluding participants who reported using thyroid-related drugs in their prescription medication records. Thyroid-related medications were identified by matching the drug identifier RXDDRGID from the NHANES prescription drug file with the drug classification file. Drugs classified as thyroid hormones or antithyroid agents according to the Multum therapeutic classification were defined as thyroid-related medications. The excluded thyroid-related medications included levothyroxine, liothyronine, desiccated thyroid, thyroid hormones, methimazole, propylthiouracil and potassium iodide. After excluding thyroid medication users, 2,426 healthy controls (HC) and 576 individuals with HT were included. Applying the same inclusion and exclusion criteria, 401 individuals with HT and euthyroidism, 38 with HT and subclinical hypothyroidism, and 40 with HT and overt hypothyroidism were included.

### 2.2 Ethics statement

This study involving local clinical samples and data should receive approval from the relevant ethics committee, and all participants should provide informed consent. NHANES is a public database approved by the National Center for Health Statistics ethics review board, with written informed consent obtained from participants. This study are approved by the Ethics Committee of the PLA No. 967 Hospital of the Joint Support Force (approval number PLA967-GC2026-11).

### 2.3 Biological age and age acceleration

KDM biological age and PhenoAge were calculated or extracted according to published algorithms (Klemera & Doubal, 2006; Liu et al., 2018; Kwon & Belsky, 2021). This study included three independent cohorts: discovery cohort 1, discovery cohort 2, and the NHANES validation cohort. Each cohort was processed independently, with no pooled modeling, no pooled statistical analysis, and no cross-cohort imputation.

In discovery cohort 1, PhenoAge was calculated using the standard Levine PhenoAge algorithm. Variables included chronological age, albumin, creatinine, glucose, lnCRP, lymphocyte percentage, MCV, RDW, alkaline phosphatase, and WBC.

The PhenoAge linear predictor was calculated as follows:

XB = -19.907 - 0.0336 x Albumin + 0.0095 x Creatinine + 0.1953 x Glucose + 0.0954 x lnCRP - 0.0120 x Lymphocyte% + 0.0268 x MCV + 0.3306 x RDW + 0.00188 x ALP + 0.0554 x WBC + 0.0804 x Age.

Mortality = 1 - exp{-exp(XB) x [exp(120 x 0.0076927) - 1]/0.0076927}.

PhenoAge = 141.50225 + log[-0.00553 x log(1 - Mortality)]/0.090165.

Discovery cohort 1 used a sample-specific modified KDM method for KDM biological age, incorporating albumin, creatinine, glucose, lnCRP, lymphocyte percentage, MCV, RDW, ALP, and WBC. For each biomarker, a linear model of biomarker value against chronological age was fitted within the cohort:

Biomarkerj = qj + kj x Age + epsilon.

where qj is the intercept, kj is the age slope, and sj is the residual standard deviation. KDM biological age was calculated as follows:

KDM Age = {Σj[(Biomarkerj − qj) × kj/sj²] + Age/sBA²}/{Σj(kj²/sj²) + 1/sBA²}.

where sBA^2^ was set in the code as the variance of chronological age in the current training sample. The KDM model in discovery cohort 1 included lnCRP.

In discovery cohort 2, because CRP was unavailable, PhenoAge, PhenoAge acceleration, and PhenoAge accelerated status were not calculated. Only KDM biological age was calculated. The same sample-specific modified KDM method as in discovery cohort 1 was used, but without CRP/lnCRP. Biomarkers included albumin, creatinine, glucose, lymphocyte percentage, MCV, RDW, ALP, and WBC.

In the NHANES validation cohort, PhenoAge and KDM biological age were calculated using the BioAge package. The included indicators were albumin, ALP, BUN, creatinine, CRP, HbA1c, lnCRP, total cholesterol, glucose, lymphocyte percentage, MCV, RDW, WBC, FEV1, and SBP.

Age acceleration was defined as follows in all three cohorts: PhenoAge acceleration = PhenoAge - chronological age; KDM age acceleration = KDM age - chronological age. Age acceleration status was defined as an acceleration value >0: accelerated = 1 if acceleration >0; accelerated = 0 if acceleration <=0. Additional cohort-specific details are provided in **Supplementary Material 3**.

### 2.4 Metabolomics

Serum samples were analyzed by liquid chromatography-mass spectrometry (LC-MS). Metabolite detection was performed using an LC-MS full-component data acquisition system, with chromatographic separation and mass spectrometry data acquisition conducted in positive and negative ion modes. To minimize systematic error, sample order was randomized, and quality control (QC) samples were used to monitor analytical stability. QC samples were prepared by pooling equal volumes of all study samples, used for system equilibration before formal injection, and then inserted after every 10 study samples.

In positive ion mode, the chromatographic column was a Waters BEH C8 column (50 mm x 2.1 mm, 1.7 μm). The column temperature was 60 °C and the flow rate was 0.4 ml/min. Mobile phase A was water containing 0.1% formic acid, and mobile phase B was acetonitrile containing 0.1% formic acid. The gradient elution program was as follows: initial B ratio 5% for 0.5 min; linear increase to 40% over 1.5 min; linear increase to 100% over 6 min and maintained for 2 min; returned to 5% B at 10.1 min and equilibrated for 2 min.

In negative ion mode, the chromatographic column was an ACQUITY UPLC HSS T3 column (50 mm x 2.1 mm, 1.8 μm). The column temperature was 60 °C and the flow rate was 0.4 ml/min. Mobile phase A was water containing 6.5 mM NH4HCO3, and mobile phase B was 95% methanol containing 6.5 mM NH4HCO3. The gradient elution program was as follows: initial B ratio 2% for 0.5 min; increase to 40% over 2 min; linear increase to 100% over 6 min and maintained for 2 min; returned to 2% B at 10.1 min and equilibrated for 1.9 min.

Mass spectrometry was performed in full-scan mode. Scan ranges were m/z 80–1200 in both positive and negative ion modes, with spray voltages of 3.50 kV and 3.00 kV, respectively. Capillary temperature was 300 °C, auxiliary gas heater temperature was 350 °C, sheath gas and auxiliary gas flow rates were 45 and 10 arbitrary units, and mass resolution was 7 x 10^4.

Raw LC-MS data were subjected to peak extraction, peak alignment, peak matching, and peak area integration. Metabolite features were corrected by internal standards and normalized to total peak area. For quality control, features with coefficient of variation (CV) >30% in QC samples and features detected in <80% of samples in any group were removed. Metabolites with >20% missing values were excluded; remaining missing values were imputed by KNN. Data were then log2-transformed and z-score standardized before statistical analyses and metabolic age model construction.

Metabolite annotation and identification confidence were assessed based on the LC−MS annotation report. Metabolite annotation primarily relied on ionization mode, retention time, calculated m/z, molecular formula, mass error, database matching score and available database identifiers.

For the three prioritized core metabolites, citric acid was annotated in negative ion mode with retention time (RT) = 3.088 min, calculated m/z = 191.01987, molecular formula C_6_H_8_O_7_, mass error = 1.247 ppm and a database matching score of 88.04. According to the metabolite annotation report, citric acid was assigned a Level 2 annotation. LPC 20:0 (sn−1) and SM 34:2 were annotated as lipid metabolites based on their corresponding LC−MS annotation records.

### 2.5 Metabolic age model construction

For metabolomic age construction, all participants who passed LC−MS quality control were included, comprising 77 CON, 48 EHT and 36 DHT cases.

After metabolomics data QC, batch correction, standardization, and missing value handling, healthy controls were used as the training set to construct a metabolic age prediction model. Chronological age was the dependent variable and the processed metabolite abundance matrix was the independent variable; a random forest regression algorithm was used to establish the metabolic age model.

Internal model performance was assessed by five-fold cross-validation. Healthy controls were randomly divided into five subsets; each time, four subsets were used for model training and the remaining subset for validation. Predictions from the five repeats were summarized. Model performance was evaluated using RMSE, MAE, Pearson R, and R2. After cross-validation, the final metabolic age model was trained using all healthy controls and applied to CON, EHT, and DHT participants to obtain predicted metabolic age for each subject. MAA was defined as predicted metabolic age minus chronological age:

MAA = predicted metabolic age - chronological age.

MAA >0 was defined as metabolic age acceleration, while MAA <=0 was defined as no metabolic age acceleration. Group differences in MAA among CON, EHT, and DHT were then compared, followed by analyses of correlations between MAA and clinical indicators.

### 2.6 Candidate metabolite screening and predictive model inclusion

For candidate metabolite screening and thyroid function prediction modeling, a subset of participants from the metabolomic age construction cohort with strictly complete clinical data was further selected for analysis, including 35 CON, 30 EHT and 32 DHT cases.

After metabolomics preprocessing, metabolite abundance data were converted to numeric values, missing values were imputed with half of the nonzero minimum value of each metabolite, and log2(x + 1) transformation was applied. Correlations between each metabolite and age, TSH, FT3, and FT4 were analyzed. Pearson correlation analysis was used, and metabolites with P <0.05 were considered significantly correlated and classified as positively or negatively correlated according to the direction of correlation. Differential metabolite analyses were then conducted for EHT vs CON and DHT vs CON. Wilcoxon rank-sum tests were used for two-group comparisons, with P <0.05 as the screening threshold. Fold change was calculated from the difference in mean abundance after log2 transformation, i.e., FC = 2^(mean disease - mean control). FC >1 indicated upregulation and FC <1 indicated downregulation.

Candidate metabolites were screened using an intersection strategy. Significant differential metabolites from EHT vs CON and DHT vs CON were extracted and intersected to obtain metabolites significantly changed in both EHT and DHT. Metabolites significantly correlated with at least one clinical indicator (age, TSH, FT3, or FT4) were then extracted. Final candidate metabolites were defined as metabolites satisfying both conditions: (1) significant differential abundance in both EHT vs CON and DHT vs CON comparisons; and (2) significant correlation with at least one of age, TSH, FT3, or FT4.

For candidate metabolites, correlation coefficients, correlation P values, differential directions, FC values, differential analysis P values, and metabolite classes were compiled and exported as a summary table. To visualize the screening process, UpSet plots were used to display intersections among metabolite sets from EHT vs CON, DHT vs CON, age-related, TSH-related, FT3-related, and FT4-related metabolites; Venn diagrams were used to display intersections among EHT differential metabolites, DHT differential metabolites, and clinical phenotype-related metabolites. KEGG pathway enrichment analysis was performed using MetaboAnalyst 6.0, with significance set at P <0.05.

Based on the candidate metabolites, HT classification models and TSH, FT3, and FT4 prediction models were constructed. The modeling dataset included candidate metabolites, age, and sex. Sex was converted into a binary variable, with male = 1 and female = 0. For the HT classification model, controls were coded as 0, and EHT and DHT participants were combined as the HT disease group and coded as 1. Final predictors included age, sex, and candidate metabolites.

Models were constructed using the glmnet package. The HT classification model used binomial logistic regression, and the TSH, FT3, and FT4 prediction models used Gaussian regression. All predictors were standardized before modeling. Ridge regression regularization was used for all models (alpha = 0), and the optimal regularization parameter lambda.min was selected by five-fold cross-validation. The random seed was set to 123 to ensure reproducibility.

The predictive performance of the HT classification model was evaluated using ROC curves and AUC, and Wilcoxon rank-sum tests were used to compare predicted probabilities between disease and control groups. For TSH, FT3, and FT4 regression models, performance was evaluated by Pearson r and P values between predicted and measured values. Feature contribution was shown using regression coefficients at lambda.min, and variables ranking among the top 14 by absolute coefficient value were visualized. Coefficient results for all models were exported as CSV files.

### 2.7 Sensitivity analysis

Considering that some participants might be currently using or have previously used thyroid−related medications, we performed a sensitivity analysis by excluding thyroid medication users and re−examined the associations between HT and biological age as well as age acceleration metrics. This analysis tested whether the observed associations were primarily driven by thyroid−related treatments.

Furthermore, because the biomarkers included in the construction of KDM age and PhenoAge were not completely consistent across cohorts, particularly with respect to the availability of CRP−related indicators, we additionally reconstructed KDM age and PhenoAge without CRP in both Cohort 1 and the NHANES validation cohort. Using these no−CRP biological age metrics, we repeated the between−group comparisons and regression analyses to assess the robustness and cross−cohort consistency of the associations between HT and biological age acceleration.

### 2.8 Statistical analysis

Continuous variables were reported as mean ± standard deviation or median [interquartile range] according to distribution; categorical variables as counts and percentages, n (%). Two-group comparisons of continuous variables used Wilcoxon rank-sum tests; categorical variables used Pearson chi-square tests or Fisher’s exact tests. Multi-group comparisons of continuous variables used Kruskal-Wallis tests. Baseline characteristic tables were generated using gtsummary and flextable.

Continuous biological age indicators were analyzed using multivariable linear regression models, and age acceleration status was analyzed using logistic regression models. Results are expressed as regression coefficients beta, odds ratios (ORs), and 95% confidence intervals. Primary models in discovery cohort 1, discovery cohort 2, and the omics cohort were adjusted for age and sex. In NHANES, model 1 was adjusted for age and sex; model 2 further adjusted for race/ethnicity, poverty-income ratio, education, alcohol intake, smoking, and physical activity. Primary regression models for metabolomics were adjusted for age and sex.

Metabolite correlations with age, TSH, FT3, and FT4 were analyzed by Pearson correlation, with P <0.05 as the screening threshold. Differential metabolites in EHT vs CON and DHT vs CON were compared by Wilcoxon rank-sum tests with P <0.05. For prediction models, the HT classification model used regularized logistic regression, whereas TSH, FT3, and FT4 prediction models used regularized linear regression; all were implemented with glmnet. The regularization parameter was determined by five-fold cross-validation. Classification model performance was evaluated using ROC curves and AUC, and regression model performance by Pearson correlations and corresponding P values between predicted and measured values.

PCA was performed using prcomp, and PLS-DA using ropls. Correlation heatmaps were plotted using Hmisc, ComplexHeatmap, and circlize. Venn and UpSet plots were generated using ggVennDiagram, VennDiagram, and UpSetR. All statistical tests were two-sided, and P <0.05 was considered statistically significant.

All statistical analyses were conducted in R version 4.3.3. The main R packages used included ggplot2, dplyr, tidyr, openxlsx, gtsummary, flextable, glmnet, pROC, caret, Hmisc, ComplexHeatmap, circlize, UpSetR, ggVennDiagram, VennDiagram, and ropls.

## 3 Results

### 3.1 Baseline characteristics

This study included four data sources: discovery cohort 1 (249 healthy controls and 159 HT patients), discovery cohort 2 (27 healthy controls and 31 HT patients), the metabolomics cohort (77 CON, 48 EHT, and 36 DHT participants), and the NHANES 2007–2012 validation cohort (overall n = 3,188, including 2,483 healthy controls and 705 HT patients).

Compared with healthy controls, the HT group in discovery cohort 1 differed in age, sex (female predominance), TSH, FT3, TgAb, TPOAb, CRP, HbA1c, TC, albumin, glucose, lymphocyte percentage, MCV, ALP, and WBC. In discovery cohort 2, the HT group differed in TSH, TgAb, TPOAb, HbA1c, and WBC. In the NHANES validation cohort, the HT group differed from healthy controls in age, sex (female predominance), TSH, FT3, TT3, TT4, TPOAb, albumin, ALP, BUN, CRP, HbA1c, glucose, and RDW. Baseline characteristics for each cohort are shown in **Tables 1**–**5**; **Supplementary Material 1**.

**Table 1:** Baseline clinical characteristics and biological age indicators in discovery cohort 1.

Characteristic	Healthy control n = 2491	Hashimoto’s thyroiditis n = 1591	P-value^2^
Age (years)	69.00 [62.00, 76.00]	72.00 [66.00, 80.00]	<0.001
Gender			<0.001
Female	121 (48.6%)	116 (73.0%)	
Male	128 (51.4%)	43 (27.0%)	
TSH (μIU/ml)	1.50 [1.00, 2.32]	2.01 [1.02, 3.72]	<0.001
FT3 (pmol/l)	4.05 [3.57, 4.53]	3.60 [2.74, 4.14]	<0.001
FT4 (pmol/l)	16.00 [14.50, 17.50]	15.20 [13.55, 17.50]	0.032
TGAb (IU/ml)	19.90 [18.00, 22.10]	217.00 [53.00, 426.00]	<0.001
TPOAb (IU/ml)	11.20 [9.00, 14.20]	84.70 [31.50, 196.00]	<0.001
CRP (mg/L)	2.30 [1.40, 3.90]	4.45 [1.60, 37.57]	<0.001
HbA1c (%)	5.70 [5.40, 6.00]	6.20 [5.65, 7.40]	<0.001
TC (mmol/L)	3.90 [3.33, 4.68]	3.68 [2.75, 4.49]	0.015
Albumin (g/L)	40.70 [37.77, 43.42]	41.40 [39.20, 44.10]	<0.001
Creatinine (μmol/L)	68.00 [57.00, 82.00]	68.00 [59.00, 80.40]	0.18
Glucose (mmol/L)	6.65 [6.17, 7.45]	6.49 [6.02, 6.97]	<0.001
Lymphocyte percent (%)	27.60 [22.10, 35.20]	26.30 [15.20, 33.70]	<0.001
MCV (fL)	92.40 [89.90, 94.90]	92.30 [88.65, 95.85]	0.011
RDW (%)	13.21 [12.73, 13.76]	13.37 [12.90, 14.41]	0.852
ALP (U/L)	68.00 [58.00, 82.00]	76.00 [61.50, 92.00]	0.027
WBC (10^9/L)	5.82 [4.66, 7.03]	6.01 [5.05, 8.12]	0.004
PhenoAge (years)	64.85 [56.46, 75.57]	75.16 [63.22, 88.93]	<0.001
PhenoAge Acceleration (years)	-4.05 [-7.25, 0.44]	1.52 [-4.30, 10.78]	<0.001
KDM Age (years)	63.00 [55.61, 72.10]	70.31 [61.72, 81.02]	<0.001
KDMAge Acceleration (years)	-4.81 [-7.57, -0.64]	-2.32 [-6.57, 5.05]	<0.001
PhenoAge Accelerated (%)	68 (27.3%)	87 (57.2%)	<0.001
KDMAge Accelerated (%)	48 (19.3%)	58 (38.2%)	<0.001
No -crp KDM Age (years)	64.74 [57.23, 73.99]	71.60 [63.54, 80.90]	<0.001
No -crp KDMAge Acceleration (years)	-3.52 [-5.97, 0.08]	-1.41 [-5.12, 4.34]	<0.001
No -crp KDMAge Accelerated (%)	66 (26.5%)	65 (40.9%)	0.002
No -crp PhenoAge (years)	66.33 [58.18, 77.93]	76.32 [66.07, 89.47]	<0.001
No -crp PhenoAge Acceleration (years)	-2.15 [-5.37, 1.61]	2.75 [-2.32, 11.08]	<0.001
No -crp PhenoAge Accelerated (%)	85/249 (34.1%)	97/159 (61.0%)	<0.001
1 Median (Q1, Q3); n (%) 2 Pearson chi-square test or Shapiro-Wilk normality first; Wilcoxon rank-sum test	1 Median (Q1, Q3); n (%) 2 Pearson chi-square test or Shapiro-Wilk normality first; Wilcoxon rank-sum test	1 Median (Q1, Q3); n (%) 2 Pearson chi-square test or Shapiro-Wilk normality first; Wilcoxon rank-sum test	1 Median (Q1, Q3); n (%) 2 Pearson chi-square test or Shapiro-Wilk normality first; Wilcoxon rank-sum test

TSH, thyroid-stimulating hormone (0.27-4.2 μIU/ml); FT3, free triiodothyronine (3.1-6.8 pmol/L); FT4, free thyroxine (12–22 pmol/L); TGAb, thyroglobulin antibody (<115 IU/mL); TPOAb, thyroid peroxidase antibody (<34 IU/mL); CRP, C-reactive protein (<6 mg/L); HbA1c, glycated hemoglobin (4.0%-6.0%); TC, total cholesterol (3.1-5.7 mmol/L); albumin (40–55 g/L); creatinine (45-104 μmol/L); glucose (3.9-6.11 mmol/L); lymphocyte percentage (20%-50%); MCV, mean corpuscular volume (82–100 fL); RDW, red cell distribution width (37%-54%); ALP, alkaline phosphatase (38–126 U/L); WBC, white blood cell count (3.5-9.5 x 10^9/L).

**Table 2:** Baseline clinical characteristics and biological age indicators in discovery cohort 2.

Characteristic	Healthy control n = 271	Hashimoto’s thyroiditis n = 311	P-value^2^
Age (years)	54.00 [42.00, 61.00]	61.00 [41.00, 67.00]	0.4
Gender			>0.9
Female	23/27 (85.2%)	27/31 (87.1%)	
Male	4/27 (14.8%)	4/31 (12.9%)	
TSH (μIU/ml)	1.14 [0.80, 1.98]	1.62 [1.04, 2.31]	0.045
FT3 (pmol/l)	4.93 [4.04, 5.90]	4.78 [4.50, 5.56]	0.2
FT4 (pmol/l)	14.57 ± 3.66	14.93 ± 3.42	0.2
TPOAb (%)	10.38 [9.50, 10.94]	50.14 [37.47, 67.48]	<0.001
TGAb (%)	8.52 ± 0.97	39.84 ± 11.37	<0.001
Albumin (g/L)	44.19 ± 2.29	43.77 ± 2.26	0.489
Creatinine (μmol/L)	50.00 [46.30, 56.05]	52.90 [46.50, 58.90]	0.523
Glucose (mmol/L)	5.20 [5.05, 5.40]	5.60 [5.10, 6.01]	0.028
Lymphocyte percent (%)	34.61 ± 7.61	34.90 ± 8.79	0.894
MCV (fL)	90.40 [87.90, 93.30]	92.40 [89.35, 93.75]	0.105
RDW (%)	13.59 [13.23, 13.85]	12.91 [12.49, 13.36]	0.001
ALP (U/L)	65.81 ± 11.86	76.35 ± 26.33	0.051
WBC (10^9/L)	5.81 ± 1.14	5.86 ± 1.68	0.889
HbA1c (%)	5.50 [5.25, 5.60]	5.70 [5.50, 5.95]	0.006
Urea (mmol/L)	4.90 [4.25, 5.40]	5.00 [3.95, 5.70]	0.685
SBP (mmHg)	122.22 ± 13.78	132.14 ± 22.41	0.05
TC (mmol/L)	4.46 ± 0.50	5.35 ± 1.03	<0.001
KDM Age (years)	47.88 [40.31, 55.18]	61.21 [42.04, 67.92]	0.019
KDMAge Acceleration (years)	-3.56 ± 5.72	3.10 ± 6.92	<0.001
KDMAge Accelerated (%)	9/27 (33.3%)	21/31 (67.7%)	0.009
1 Median (Q1, Q3); n (%) 2 Wilcoxon rank sum test; Fisher’s exact test; Wilcoxon rank sum exact test.	1 Median (Q1, Q3); n (%) 2 Wilcoxon rank sum test; Fisher’s exact test; Wilcoxon rank sum exact test.	1 Median (Q1, Q3); n (%) 2 Wilcoxon rank sum test; Fisher’s exact test; Wilcoxon rank sum exact test.	1 Median (Q1, Q3); n (%) 2 Wilcoxon rank sum test; Fisher’s exact test; Wilcoxon rank sum exact test.

TSH, thyroid-stimulating hormone (0.25-4 μIU/ml); FT3, free triiodothyronine (2.91-9.08 pmol/L); FT4, free thyroxine (9.05-25.5 pmol/L); TMAb, thyroid microsomal antibody (<20%); TGAb, thyroglobulin antibody (<30%); albumin (40–55 g/L); creatinine (59-97 μmol/L); glucose (3.9-6.11 mmol/L); lymphocyte percentage (20%-50%); MCV, mean corpuscular volume (82–100 fL); RDW, red cell distribution width (37%-50%); ALP, alkaline phosphatase (45–125 U/L); WBC, white blood cell count (3.5-9.5 x 10^9/L); HbA1c, glycated hemoglobin (4%-6%); urea (3.1–8 mmol/L); SBP (<140 mmHg); TC, total cholesterol (3.1-5.17 mmol/L).

**Table 3:** Baseline clinical characteristics and biological age indicators in the NHANES validation cohort.

Characteristic	Healthy control n=2483	Hashimoto’s thyroiditis n=705	p
Age (years)	39.00 [29.00, 52.00]	52.00 [39.00, 64.00]	<0.001
Gender (%)			<0.001
Female	1190 (47.9%)	463 (65.7%)	
Male	1293 (52.1%)	242 (34.3%)	
Race (%)			0.003
Mexican American	424 (17.1%)	150 (21.3%)	
Non-Hispanic Black	408 (16.4%)	79 (11.2%)	
Non-Hispanic White	1271 (51.2%)	364 (51.6%)	
Other	107 (4.3%)	27 (3.8%)	
Other Hispanic	273 (11.0%)	85 (12.1%)	
PIR level (%)			0.122
Below poverty	514 (20.7%)	124 (17.6%)	
High income	707 (28.5%)	204 (28.9%)	
Low income	615 (24.8%)	167 (23.7%)	
Middle income	647 (26.1%)	210 (29.8%)	
Education level (%)			0.159
college or above	1306 (52.6%)	347 (49.2%)	
high school or equivalent	590 (23.8%)	168 (23.8%)	
less than high school	587 (23.6%)	190 (27.0%)	
Drinking (%)			0.005
heavy drinker	267 (10.8%)	62 (8.8%)	
low to moderate drinker	697 (28.1%)	241 (34.2%)	
non-drinker	1519 (61.2%)	402 (57.0%)	
Smoking status (%)			<0.001
Current smoker	622 (25.1%)	129 (18.3%)	
Former smoker	514 (20.7%)	185 (26.2%)	
Never smoker	1347 (54.2%)	391 (55.5%)	
Physical activity (%)			0.013
High physical activity	1848 (74.4%)	486 (68.9%)	
Low physical activity	338 (13.6%)	121 (17.2%)	
Middle physical activity	297 (12.0%)	98 (13.9%)	
FT3 (pg/ml)	3.20 [3.00, 3.49]	3.10 [2.83, 3.30]	<0.001
TT3 (ng/dL)	113.00 [101.00, 128.00]	109.00 [96.00, 124.00]	<0.001
FT4 (pmol/L)	10.30 [9.00, 10.70]	10.30 [9.00, 11.60]	0.324
TT4 (ug/dL)	7.60 [6.80, 8.60]	7.70 [6.80, 8.90]	0.167
TSH (mIU/L)	1.47 [1.02, 2.09]	2.16 [1.23, 3.35]	<0.001
TPOAB (IU/mL)	0.60 [0.30, 1.10]	43.20 [11.80, 195.40]	<0.001
TGAB (IU/mL)	0.60 [0.60, 0.60]	3.00 [0.60, 16.80]	<0.001
Albumin (g/L)	43.00 [41.00, 45.00]	42.00 [40.00, 44.00]	<0.001
ALP (IU/L)	64.00 [52.00, 77.00]	66.00 [55.00, 80.00]	0.003
BUN (mg/dl)	11.00 [9.00, 14.00]	12.00 [10.00, 15.00]	<0.001
Serum Creatinine(μmol/L)	72.49 [63.65, 85.75]	72.49 [60.11, 81.33]	0.003
CRP (mg/dL)	0.14 [0.06, 0.33]	0.19 [0.08, 0.45]	<0.001
HbA1c (%)	5.30 [5.10, 5.50]	5.60 [5.30, 5.90]	<0.001
TC (mg/dL)	192.00 [167.00, 220.00]	197.00 [172.00, 226.00]	0.003
Glucose (mmol)	4.83 [4.50, 5.11]	5.16 [4.77, 5.83]	<0.001
Lymphocyte percent (%)	30.50 [25.80, 35.70]	30.30 [25.90, 35.20]	0.731
MCV (fl)	89.20 [86.30, 92.10]	89.60 [86.40, 92.40]	0.376
RDW (%)	12.50 [12.10, 13.00]	12.60 [12.20, 13.30]	<0.001
WBC (x1000 cells)	6.90 [5.60, 8.20]	6.90 [5.70, 8.20]	0.613
FEV1 (mL)	3267.00 [2752.50, 3903.00]	2739.00 [2172.00, 3443.00]	<0.001
SBP (mmHg)	118.00 [110.00, 128.00]	122.00 [112.00, 134.00]	<0.001
KDM Age (years)	22.72 [1.02, 36.47]	37.81 [22.51, 53.15]	<0.001
KDMAge Acceleration (years)	-14.68 [-40.19, -5.29]	-10.01 [-21.14, -3.45]	<0.001
PhenoAge (years)	29.12 [18.62, 42.12]	42.86 [30.21, 56.76]	<0.001
PhenoAge Acceleration (years)	-10.53 [-13.88, -7.03]	-9.36 [-13.02, -5.07]	<0.001
KDMAge Accelerated (%)	287/2483 (11.6%)	103/705 (14.6%)	0.029
PhenoAge Accelerated (%)	103/2483 (4.1%)	61/705 (8.7%)	<0.001
KDMAge no CRP	36.14 [27.55, 47.74]	48.87 [37.45, 60.32]	<0.001
PhenoAge no CRP	34.45 [23.76, 46.73]	47.16 [34.80, 61.22]	<0.001
KDMAge acceleration no CRP	-2.83 [-6.08, 0.43]	-3.30 [-6.82, 0.58]	0.178
PhenoAge acceleration no CRP	-5.37 [-7.75, -2.93]	-4.84 [-7.55, -1.68]	0.001
KDMAge accelerated no CRP (%)	682 (27.5%)	208 (29.5%)	0.287
PhenoAge accelerated no CRP (%)	225 (9.1%)	113 (16.0%)	<0.001

**Table 4:** Baseline clinical characteristics and biological age indicators according to HT stage in the NHANES validation cohort.

Characteristic	Healthy control n=2483	Hashimoto’s thyroiditis euthyroidism n=480	Hashimoto’s thyroiditis subclinical hypothyroidism n=56	Hashimoto’s thyroiditis overt hypothyroidism n=45	p
Age (years)	39.00 [29.00, 52.00]	53.00 [39.00, 65.00]	58.00 [41.25, 67.00]	50.00 [37.00, 64.00]	<0.001
Gender (%)					<0.001
Female	1190 (47.9%)	309 (64.4%)	31 (55.4%)	36 (80.0%)	
Male	1293 (52.1%)	171 (35.6%)	25 (44.6%)	9 (20.0%)	
Race (%)					0.024
Mexican American	424 (17.1%)	105 (21.9%)	6 (10.7%)	9 (20.0%)	
Non-Hispanic Black	408 (16.4%)	64 (13.3%)	2 (3.6%)	6 (13.3%)	
Non-Hispanic White	1271 (51.2%)	235 (49.0%)	40 (71.4%)	25 (55.6%)	
Other	107 (4.3%)	22 (4.6%)	1 (1.8%)	0 (0.0%)	
Other Hispanic	273 (11.0%)	54 (11.2%)	7 (12.5%)	5 (11.1%)	
PIR level (%)					0.677
Below poverty	514 (20.7%)	82 (17.1%)	10 (17.9%)	9 (20.0%)	
High income	707 (28.5%)	138 (28.8%)	19 (33.9%)	11 (24.4%)	
Low income	615 (24.8%)	120 (25.0%)	11 (19.6%)	10 (22.2%)	
Middle income	647 (26.1%)	140 (29.2%)	16 (28.6%)	15 (33.3%)	
Education level (%)					0.055
college or above	1306 (52.6%)	225 (46.9%)	30 (53.6%)	28 (62.2%)	
high school or equivalent	590 (23.8%)	110 (22.9%)	13 (23.2%)	10 (22.2%)	
less than high school	587 (23.6%)	145 (30.2%)	13 (23.2%)	7 (15.6%)	
Drinking (%)					0.019
heavy drinker	267 (10.8%)	36 (7.5%)	7 (12.5%)	4 (8.9%)	
low to moderate drinker	697 (28.1%)	156 (32.5%)	19 (33.9%)	21 (46.7%)	
non-drinker	1519 (61.2%)	288 (60.0%)	30 (53.6%)	20 (44.4%)	
Smoking status (%)					0.005
Current smoker	622 (25.1%)	85 (17.7%)	10 (17.9%)	9 (20.0%)	
Former smoker	514 (20.7%)	124 (25.8%)	17 (30.4%)	13 (28.9%)	
Never smoker	1347 (54.2%)	271 (56.5%)	29 (51.8%)	23 (51.1%)	
Physical activity (%)					0.008
High physical activity	1848 (74.4%)	322 (67.1%)	38 (67.9%)	38 (84.4%)	
Low physical activity	338 (13.6%)	91 (19.0%)	8 (14.3%)	5 (11.1%)	
Middle physical activity	297 (12.0%)	67 (14.0%)	10 (17.9%)	2 (4.4%)	
FT3 (pg/ml)	3.20 [3.00, 3.49]	3.10 [2.90, 3.30]	3.01 [2.81, 3.32]	2.90 [2.65, 3.20]	<0.001
TT3 (ng/dL)	113.00 [101.00, 128.00]	109.00 [95.75, 124.00]	105.00 [95.75, 121.75]	104.00 [94.00, 114.00]	<0.001
FT4 (pmol/L)	10.30 [9.00, 10.70]	10.30 [9.00, 11.60]	10.30 [9.00, 11.43]	7.70 [6.50, 7.70]	<0.001
TT4 (ug/dL)	7.60 [6.80, 8.60]	7.80 [7.20, 9.10]	7.90 [6.80, 8.70]	5.90 [5.00, 6.90]	<0.001
TSH (mIU/L)	1.47 [1.02, 2.09]	1.85 [1.20, 2.69]	5.87 [5.00, 7.01]	9.34 [5.47, 17.90]	<0.001
TPOAB (IU/mL)	0.60 [0.30, 1.10]	32.40 [10.40, 151.82]	154.65 [30.77, 461.07]	214.40 [50.40, 389.60]	<0.001
TGAB (IU/mL)	0.60 [0.60, 0.60]	3.05 [0.60, 16.52]	3.75 [0.60, 27.17]	8.90 [0.90, 53.70]	<0.001
Albumin (g/L)	43.00 [41.00, 45.00]	42.00 [41.00, 44.00]	42.00 [39.75, 44.00]	43.00 [41.00, 44.00]	<0.001
ALP (IU/L)	64.00 [52.00, 77.00]	66.50 [55.00, 81.00]	68.50 [54.75, 77.00]	62.00 [52.00, 73.00]	0.008
BUN (mg/dl)	11.00 [9.00, 14.00]	12.00 [10.00, 15.00]	13.00 [11.00, 16.00]	12.00 [11.00, 13.00]	<0.001
Serum Creatinine(μmol/L)	72.49 [63.65, 85.75]	72.49 [60.11, 81.33]	78.68 [66.08, 89.29]	72.49 [62.77, 81.33]	0.014
CRP (mg/dL)	0.14 [0.06, 0.33]	0.18 [0.08, 0.40]	0.20 [0.08, 0.58]	0.16 [0.10, 0.49]	<0.001
HbA1c (%)	5.30 [5.10, 5.50]	5.60 [5.30, 6.00]	5.50 [5.30, 5.80]	5.40 [5.20, 5.70]	<0.001
TC (mg/dL)	192.00 [167.00, 220.00]	196.00 [170.00, 220.25]	197.50 [181.25, 226.00]	203.00 [178.00, 239.00]	0.031
Glucose (mmol)	4.83 [4.50, 5.11]	5.22 [4.77, 5.84]	5.13 [4.76, 5.72]	5.05 [4.66, 5.72]	<0.001
Lymphocyte percent (%)	30.50 [25.80, 35.70]	30.10 [25.28, 35.20]	30.50 [25.35, 34.75]	32.20 [28.60, 39.70]	0.083
MCV (fl)	89.20 [86.30, 92.10]	89.70 [86.30, 92.32]	90.60 [87.20, 93.30]	88.60 [86.00, 93.00]	0.256
WBC (x1000 cells)	6.90 [5.60, 8.20]	7.00 [5.70, 8.30]	6.75 [5.57, 8.40]	7.10 [5.90, 8.60]	0.697
RDW (%)	12.50 [12.10, 13.00]	12.60 [12.20, 13.30]	12.40 [12.10, 13.03]	12.70 [12.20, 13.80]	<0.001
FEV1 (mL)	3267.00 [2752.50, 3903.00]	2806.00 [2220.25, 3441.50]	2480.50 [2005.75, 3516.00]	2607.00 [1990.00, 3312.00]	<0.001
SBP (mmHg)	118.00 [110.00, 128.00]	122.00 [112.00, 134.00]	126.00 [113.50, 138.50]	118.00 [110.00, 132.00]	<0.001
KDM Age (years)	22.72 [1.02, 36.47]	37.72 [20.64, 54.22]	42.71 [19.66, 55.04]	38.65 [27.94, 52.73]	<0.001
KDMAge Acceleration (years)	-14.68 [-40.19, -5.29]	-9.74 [-23.41, -4.04]	-14.52 [-29.93, -2.70]	-7.26 [-18.21, -2.57]	<0.001
PhenoAge (years)	29.12 [18.62, 42.12]	43.86 [29.99, 57.67]	48.72 [31.40, 61.68]	41.73 [29.72, 53.85]	<0.001
PhenoAge Acceleration (years)	-10.53 [-13.88, -7.03]	-9.41 [-13.02, -4.82]	-9.50 [-11.53, -4.46]	-7.76 [-12.29, -6.21]	<0.001
KDMAge Accelerated (%)	287/2483 (11.6%)	67/480 (14.0%)	10/56 (17.9%)	7/45 (15.6%)	0.213
PhenoAge Accelerated (%)	103/2483 (4.1%)	44/480 (9.2%)	5/56 (8.9%)	2/45 (4.4%)	<0.001
KDMAge no CRP	36.14 [27.55, 47.74]	49.09 [37.10, 60.71]	53.18 [41.61, 61.55]	46.10 [35.48, 57.55]	<0.001
PhenoAge no CRP	34.45 [23.76, 46.73]	48.53 [34.71, 61.88]	51.37 [35.20, 63.85]	47.13 [31.77, 59.39]	<0.001
KDMAge acceleration no CRP	-2.83 [-6.08, 0.43]	-3.42 [-7.01, 0.42]	-2.96 [-6.55, 0.85]	-2.29 [-4.60, 0.51]	0.302
PhenoAge acceleration no CRP	-5.37 [-7.75, -2.93]	-4.62 [-7.62, -1.40]	-4.56 [-7.11, -2.29]	-5.14 [-7.55, -1.69]	0.004
KDMAge accelerated no CRP (%)	682/2483 (27.5%)	138/480 (28.8%)	18/56 (32.1%)	14/45 (31.1%)	0.771
PhenoAge accelerated no CRP (%)	225/2483 (9.1%)	81/480 (16.9%)	9/56 (16.1%)	8/45 (17.8%)	<0.001

**Table 5:** Clinical parameters in the HT metabolic age cohort.

Characteristic	Overall1	CON n = 771	EHT n = 481	DHT n = 361	P-value^2^
Age, mean (SD)	39.8 (11.1)	41.1 (10.8)	39.9 (12.4)	37.0 (10.0)	0.147
Gender, No. (%)					0.006
Female	135 (83.9%)	57 (74.0%)	45 (93.8%)	33 (91.7%)	
Male	26 (16.1%)	20 (26.0%)	3 (6.3%)	3 (8.3%)	
1Mean (SD); n (%)	1Mean (SD); n (%)	1Mean (SD); n (%)	1Mean (SD); n (%)	1Mean (SD); n (%)	1Mean (SD); n (%)
2Kruskal-Wallis rank sum test; Fisher’s exact test	2Kruskal-Wallis rank sum test; Fisher’s exact test	2Kruskal-Wallis rank sum test; Fisher’s exact test	2Kruskal-Wallis rank sum test; Fisher’s exact test	2Kruskal-Wallis rank sum test; Fisher’s exact test	2Kruskal-Wallis rank sum test; Fisher’s exact test

### 3.2 HT is associated with higher biological age and age acceleration

**Figure 1:**
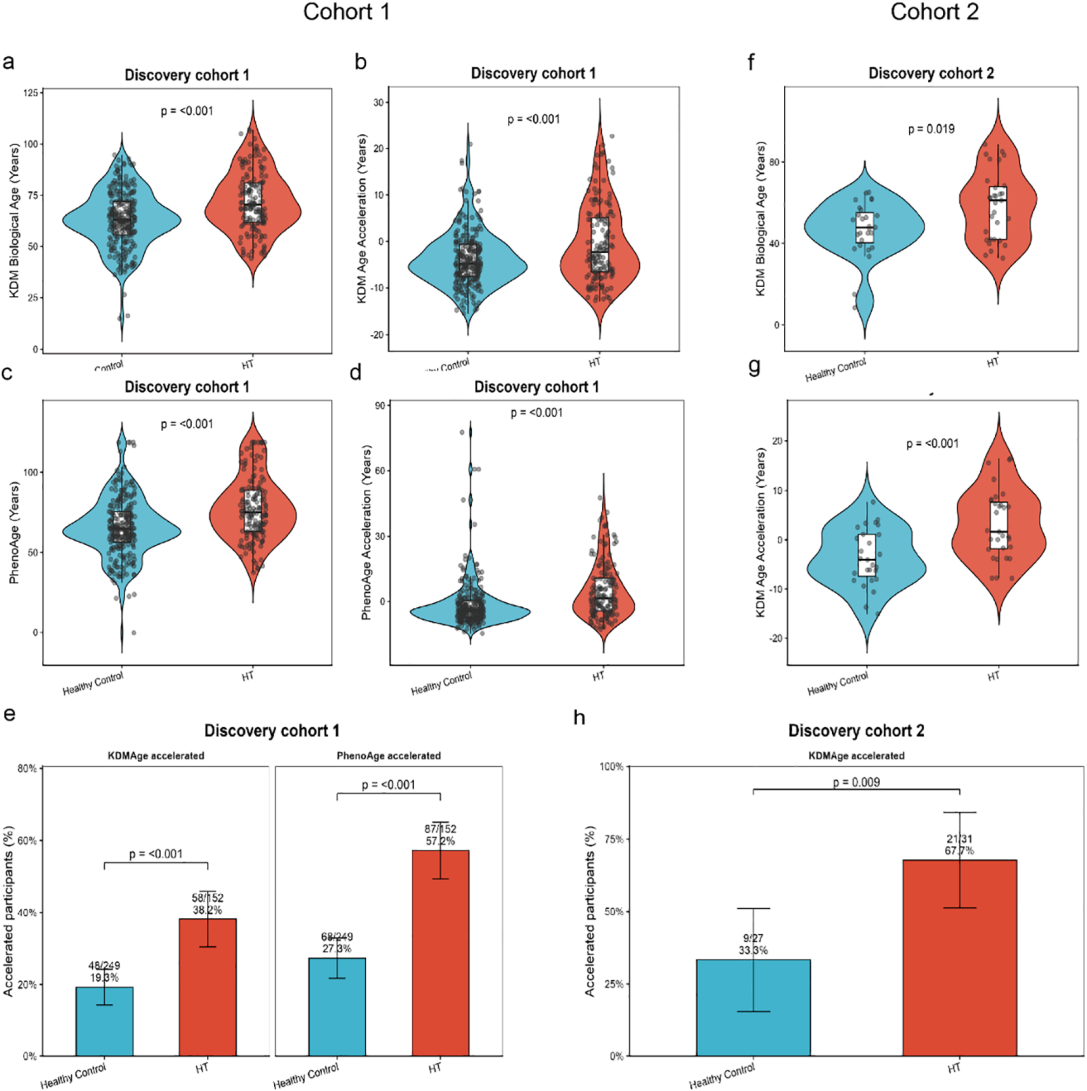
Biological age, age acceleration, and accelerated proportions in patients with HT and healthy controls in discovery cohorts 1 and 2. **(a-h)** Comparisons between healthy controls and HT patients in discovery cohort 1 for KDM biological age (KdmAge) **(a)**, KDM age acceleration **(b)**, Phenotypic Age (PhenoAge) **(c)**, PhenoAge acceleration **(d)**, and proportions of participants with KDM age or PhenoAge acceleration **(f-h)** Comparisons between healthy controls and HT patients in discovery cohort 2 for KDM biological age **(f)**, KDM age acceleration **(g)**, and the proportion of participants with KDM age acceleration **(h)**. P <0.05 was considered statistically significant.

To evaluate whether patients with HT exhibit a premature biological aging phenotype, PhenoAge, PhenoAge acceleration, KDM biological age, KDM age acceleration, and the corresponding proportions of participants with age acceleration were first calculated in discovery cohort 1. Compared with healthy controls, the HT group in discovery cohort 1 had significantly higher PhenoAge, PhenoAge acceleration, KDM biological age, KDM age acceleration, and proportions of age acceleration (**Figures 1a–e**; **Table 1**). After further adjustment for age and sex, HT remained significantly associated with higher PhenoAge (beta = 7.18 years, 95% CI 4.74-9.61), higher KDM biological age (beta = 4.28 years, 95% CI 2.87-5.69), PhenoAge accelerated status (OR = 4.17, 95% CI 2.61-6.67), and KDM accelerated status (OR = 3.74, 95% CI 2.24-6.26) (**Supplementary Table 1**). These results indicate that HT patients showed marked biological age elevation and age acceleration across two independent biological age algorithms.

In discovery cohort 2, the elevations in KDM biological age, KDM age acceleration, and the proportion of KDM age acceleration in the HT group were further validated (**Figures 1f–h**; **Table 2**). After adjustment for age and sex, HT remained significantly associated with higher KDM biological age and KDM age acceleration, both with an effect size of beta = 6.86 years (95% CI 3.38-10.33). HT was also associated with an increased likelihood of KDM accelerated status (OR = 6.32, 95% CI 1.80-22.17) (**Supplementary Table 2**). These findings further support a stable association between HT and increased biological age and age acceleration in an independent discovery cohort.

**Figure 2:**
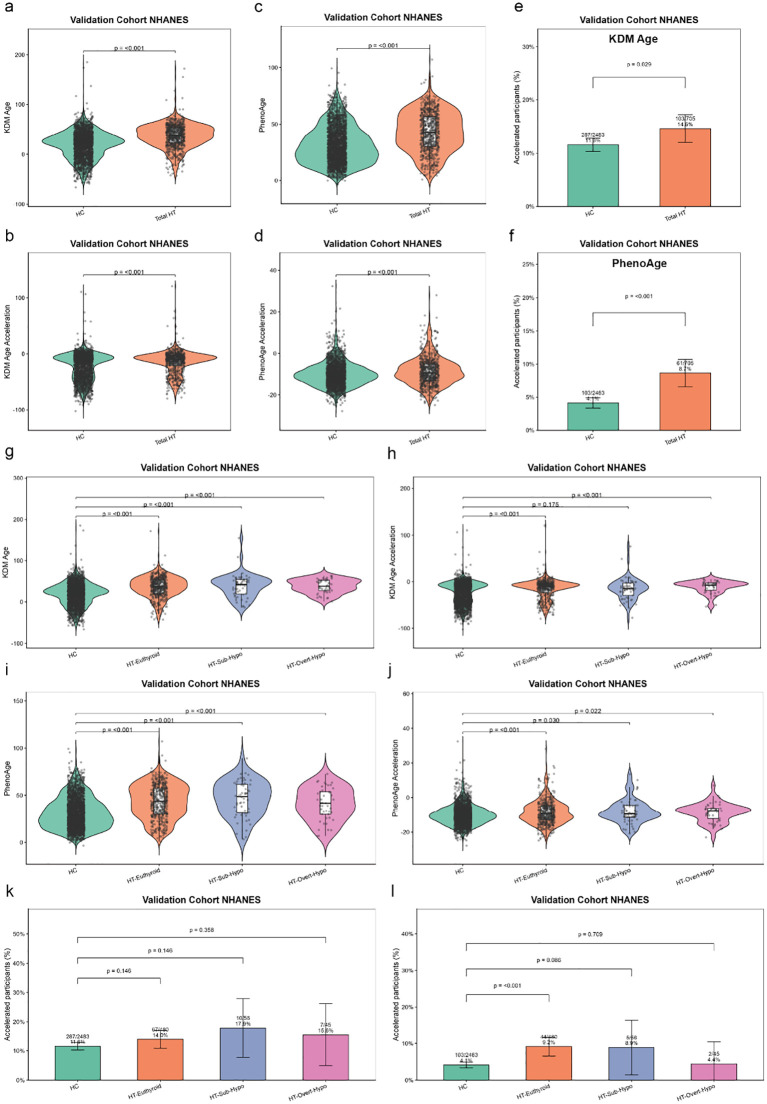
Biological age, age acceleration, and accelerated proportions in patients with HT and healthy controls in the NHANES cohort. **(a-f)** Comparisons between healthy controls and HT patients in NHANES for KDM biological age (KdmAge) **(a)**, KDM age acceleration **(b)**, PhenoAge **(c)**, PhenoAge acceleration **(d)**, the proportion with KDM age acceleration **(e)**, and the proportion with PhenoAge acceleration **(f)**. **(g-l)** Comparisons between healthy controls and HT patients at different stages in NHANES for KDM biological age **(g)**, KDM age acceleration **(h)**, PhenoAge **(i)**, PhenoAge acceleration **(j)**, the proportion with KDM age acceleration **(k)**, and the proportion with PhenoAge acceleration **(l)**. P <0.05 was considered statistically significant. HC, healthy control; HT, Hashimoto’s thyroiditis; HT-Euthyroid, Hashimoto’s thyroiditis with euthyroidism; HT-Sub-Hypo, Hashimoto’s thyroiditis with subclinical hypothyroidism; HT-Overt-Hypo, Hashimoto’s thyroiditis with overt hypothyroidism.

The association between HT and increased biological age was then examined in the NHANES 2007–2012 validation cohort. Compared with healthy controls, HT patients showed increasing trends in KDM biological age, KDM age acceleration, PhenoAge, and PhenoAge acceleration (**Figures 2a–f**; **Table 3**). After full adjustment for age, sex, race/ethnicity, poverty-income ratio, education, alcohol intake, smoking, and physical activity, overall HT remained significantly associated with higher KDM biological age and KDM age acceleration, both with an effect size of beta = 3.16 years (95% CI 1.77-4.56). HT patients were also more likely to exhibit KDM age acceleration (OR = 1.39, 95% CI 1.06-1.82). Similarly, overall HT was associated with higher PhenoAge and PhenoAge acceleration, both with an effect size of beta = 1.54 years (95% CI 1.05-2.03), and with PhenoAge accelerated status (OR = 2.29, 95% CI 1.59-3.30) (**Supplementary Table 3**). These findings suggest a stable association between HT and biological age elevation and age acceleration calculated by different algorithms in an independent population database.

To further explore the relationship between HT and biological aging across thyroid functional states, HT patients in NHANES were stratified into euthyroid HT, subclinical hypothyroid HT, and overt hypothyroid HT and compared with healthy controls. KDM biological age and PhenoAge differed among the four groups (**Figures 2g, h**; **Table 4**), with biological age generally higher in all HT subgroups than in controls. Analysis of age acceleration indicators further showed that KDM age acceleration, PhenoAge acceleration, and the proportions of KDM and PhenoAge acceleration were generally higher across HT stages than in healthy controls (**Figures 2i–l**; **Table 4**).

In multivariable regression analyses, euthyroid HT was stably associated with higher KDM biological age/KDM age acceleration; in the fully adjusted model, both effect sizes were beta = 2.71 years (95% CI 1.09-4.32). PhenoAge/PhenoAge acceleration were also significantly higher, with effect sizes of beta = 1.61 years (95% CI 1.04-2.18). Euthyroid HT was significantly associated with PhenoAge accelerated status (OR = 2.32, 95% CI 1.55-3.48), whereas the association with KDM accelerated status showed a marginally significant trend (OR = 1.34, 95% CI 0.98-1.83). Associations were more pronounced in subclinical hypothyroid HT. In the fully adjusted model, KDM biological age/KDM age acceleration were 6.22 years higher than in controls (95% CI 1.98-10.46), and PhenoAge/PhenoAge acceleration were 2.03 years higher (95% CI 0.53-3.52). Subclinical hypothyroid HT was also significantly associated with KDM accelerated status (OR = 2.29, 95% CI 1.07-4.90), while the association with PhenoAge accelerated status showed an increasing trend (OR = 2.37, 95% CI 0.90-6.23). In contrast, although overt hypothyroid HT generally showed effect directions consistent with higher biological age, most results did not reach statistical significance and had wide confidence intervals, suggesting limited statistical power due to the small sample size. Larger HT cohorts are therefore needed to validate the relationship between thyroid functional stage and biological aging.

To evaluate the influence of sex on these associations, sex-stratified analyses were performed. In both discovery and validation cohorts, the trend toward higher biological age in the HT group was generally consistent in males and females (**Supplementary Figures 1**-**S3**).

After excluding thyroid medication users, HT remained positively associated with elevated biological age and age acceleration metrics, suggesting that the relationship between HT and biological aging phenotypes is not fully explained by thyroid-related treatments. Overall, HT was positively associated with KDM age, PhenoAge and their corresponding age acceleration outcomes. Notably, the HT−Euthyroid group already showed increased biological age and a higher risk of PhenoAge acceleration, indicating that an identifiable biological aging phenotype may be present in individuals with HT even when thyroid function remains within the reference range. Sex−stratified analyses showed generally consistent direction of associations in males and females, supporting the robustness of the findings.

When the analyses were repeated using KDM age and PhenoAge metrics constructed without CRP, the positive associations between HT and elevated biological age as well as age acceleration metrics persisted. Further stratification by HT stage revealed a progressive increase in biological age and age acceleration metrics with advancing HT stage, suggesting that the HT−associated biological aging phenotype is independent of CRP inclusion and shows cross−cohort consistency.

### 3.3 HT is associated with metabolic age acceleration

To evaluate whether patients with HT exhibit age-related metabolic remodeling, a random forest metabolic age prediction model was first established using only healthy controls (CON) and internally validated by five-fold cross-validation. The model showed relatively stable age-prediction performance in the held-out test sets, with mean RMSE, MAE, R, and R2 values of 8.75, 7.11, 0.634, and 0.427, respectively; corresponding training-set metrics were 3.59, 2.83, 0.987, and 0.974 (**Supplementary Figure 4a**; **Supplementary Table 4**). These results indicate that a metabolic age model trained in healthy controls can capture age-related metabolic changes under healthy conditions. Given the difference between training- and test-set performance, the model may still be influenced by sample size, and further optimization and validation in larger samples are warranted.

**Figure 3:**
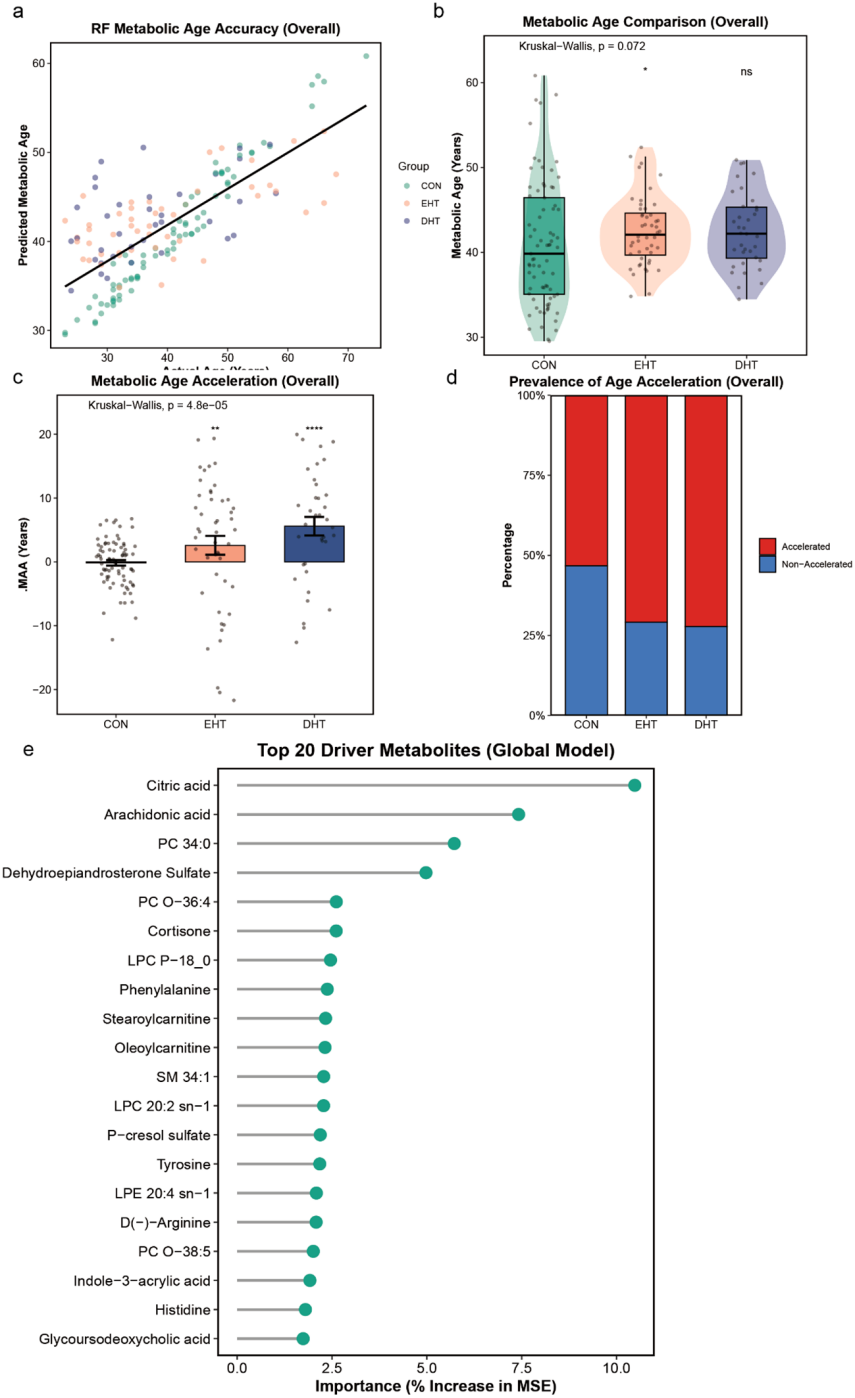
Metabolic age features, metabolic age acceleration, and key driving metabolites in patients with HT. **(a)** Prediction performance of the random forest metabolic age model, trained in healthy controls (CON) and validated by five-fold cross-validation, when applied to all samples. The x-axis represents chronological age and the y-axis represents predicted metabolic age. Each point represents one participant, colored by CON, EHT, or DHT. The black line indicates a linear fitted trend. The plot shows predictions from the final model applied to all participants. **(b)** Distribution of metabolic age across groups in the overall sample. Metabolic age distributions were compared among CON, EHT, and DHT. The violin plots show density distributions and boxplots show medians and interquartile ranges. Overall differences were assessed using the Kruskal-Wallis test; metabolic age did not differ significantly among the three groups but showed a borderline trend (P = 0.066). Symbols above the plot indicate pairwise comparisons; * indicates P <0.05 and ns indicates P >=0.05. **(c)** Distribution of metabolic age acceleration (MAA) across groups in the overall sample. MAA differed significantly among CON, EHT, and DHT (Kruskal-Wallis P = 6.6 x 10^-5). Unadjusted pairwise comparisons showed that both EHT and DHT were higher than CON. Age- and sex-adjusted effects were: EHT vs CON, beta = 2.03 (95% CI 0.66-3.41, P = 0.0041); DHT vs CON, beta = 3.35 (95% CI 1.83-4.86, P = 2.32 x 10^-5). **(d)** Proportion of participants with metabolic age acceleration across groups in the overall sample. Stacked bar charts show the distribution of accelerated and non-accelerated metabolic age status in CON, EHT, and DHT. Red indicates accelerated and blue indicates non-accelerated status. EHT and DHT had higher proportions of metabolic age acceleration than CON, with the highest proportion in DHT. **(e)** Top 20 driving metabolites ranked by variable importance from the final CON-trained model. Variable importance was assessed using the random forest model and shown as percentage increase in mean squared error (MSE). LPC, lysophosphatidylcholine; PC, phosphatidylcholine; LPE, lysophosphatidylethanolamine; FFA, free fatty acid.

After internal validation, the final metabolic age model was trained using all CON samples and applied to all participants to obtain predicted metabolic age. Predicted metabolic age was positively correlated with chronological age overall (**Figure 3a**), indicating that the healthy control-based metabolic age model captured age-related metabolic features across all samples. When predicted metabolic age distributions were compared among CON, EHT, and DHT groups, no statistically significant difference in absolute metabolic age was observed, although a borderline trend was present (Kruskal-Wallis P = 0.066; **Figure 3b**; **Supplementary Table 5**). Unadjusted pairwise comparisons showed that predicted metabolic age was higher in EHT than in CON (P = 0.046), whereas differences between DHT and CON or between EHT and DHT were not statistically significant.

Metabolic age acceleration (MAA) was then defined as predicted metabolic age minus chronological age to evaluate whether an individual’s metabolic state was advanced or delayed relative to chronological age. MAA differed significantly among CON, EHT, and DHT groups (Kruskal-Wallis P = 6.6 x 10^-5; **Figure 3c**; **Supplementary Table 6**). Unadjusted pairwise comparisons showed that MAA was significantly higher in both EHT and DHT than in CON (P = 0.0046 and P = 3.1 x 10^-5, respectively), whereas EHT and DHT did not differ significantly (P = 0.2289). After further adjustment for age and sex, the association between HT and higher MAA remained stable. Compared with CON, MAA increased by 2.03 units in EHT (beta = 2.03, 95% CI 0.66-3.41, P = 0.0041) and by 3.35 units in DHT (beta = 3.35, 95% CI 1.83-4.86, P = 2.32 x 10^-5). These results suggest that HT patients exhibit metabolic age acceleration and that acceleration may be more pronounced in HT patients with thyroid dysfunction.

Further analysis of the proportion of participants with metabolic age acceleration showed that both EHT and DHT had higher proportions of accelerated individuals than CON (**Figure 3d**), further supporting a premature metabolic aging phenotype in HT. Sex-stratified analyses showed that the increasing trend in MAA among EHT and DHT patients was broadly consistent in females and males (**Supplementary Figure 4**), suggesting a degree of consistency in HT-related metabolic age acceleration across sexes. To identify key metabolites driving metabolic age prediction, variable importance was calculated using the global random forest model and ranked by percentage increase in MSE. Citric acid had the highest variable importance, followed by arachidonic acid and dehydroepiandrosterone sulfate. Indole-3-acrylic acid, LPC 20:2 sn-1, PC 34:0, L-kynurenine, myo-inositol, glutamine, tyrosine, and multiple lipid molecules also ranked among the top 20 (**Figure 3e**; **Supplementary Table 7**). These findings suggest that abnormalities related to energy metabolism, lipid metabolism, amino acid metabolism, and steroid metabolism may jointly participate in HT-related metabolic age remodeling.

### 3.4 HT-related metabolic fluctuations and core marker identification

To further dissect HT-related metabolic abnormalities and identify potential core metabolic markers, participants with complete clinical data were selected for analysis, including 35 CON, 30 EHT, and 32 DHT participants. Baseline characteristics are shown in **Table 6**. PCA and PLS-DA showed partial separation among the three groups at the overall metabolomic profile level, suggesting systemic metabolic remodeling in HT patients (**Supplementary Figure 5**).

**Table 6:** Clinical parameters in the HT metabolomics cohort.

Characteristic	CON n = 35	EHT n = 30	DHT n = 32	P-value^1^
Gender, n (%)				<0.001
Female	22 (63%)	29 (97%)	29 (91%)	
Male	13 (37%)	1 (3.3%)	3 (9.4%)	
Age (years), Mean (SD)	38 (11)	43 (13)	37 (10)	0.087
TSH (mIU/L), Mean (SD)	1.7 (0.8)	2.2 (1.0)	22.0 (40.9)	<0.001
FT3 (pmol/L), Mean (SD)	5.10 (0.49)	4.85 (0.44)	4.97 (2.10)	0.042
FT4 (pmol/L), Mean (SD)	15.85 (2.07)	15.21 (2.45)	12.63 (3.81)	<0.001
TgAb (U/mL), Mean (SD)	16 (6)	222 (153)	272 (171)	<0.001
TPOAb (U/mL), Mean (SD)	31 (6)	897 (525)	1,008 (490)	<0.001
1Pearson’s Chi-squared test; Kruskal-Wallis rank sum test	1Pearson’s Chi-squared test; Kruskal-Wallis rank sum test	1Pearson’s Chi-squared test; Kruskal-Wallis rank sum test	1Pearson’s Chi-squared test; Kruskal-Wallis rank sum test	1Pearson’s Chi-squared test; Kruskal-Wallis rank sum test

FT3, free triiodothyronine; TT3, total triiodothyronine; FT4, free thyroxine; TT4, total thyroxine; TSH, thyroid stimulating hormone; TPOAB, thyroid peroxidase antibody; TGAB, thyroglobulin antibody; ALP, alkaline phosphatase; BUN, blood urea nitrogen; CRP, C-reactive protein; HbA1c,glycated hemoglobin A1c; TC, total cholesterol; MCV, mean corpuscular volume; RDW, red cell distribution width; WBC, white blood cell count; FEV1, forced expiratory volume in 1 second; SBP, systolic blood pressure; CON, Control; HC, healthy controls; HT, Hashimoto’s thyroiditis; EHT, Euthyroid Hashimoto’s thyroiditis; DHT, Overt hypothyroid Hashimoto’s thyroiditis; KDM biological age, Klemera-Doubal method biological age; PhenoAge, Phenotypic Age; MAA, Metabolic age acceleration; LPC, Lysophosphatidylcholine; SM, Sphingomyelin; PC, phosphatidylcholine; TCA, Tricarboxylic Acid Cycle; ATP, Adenosine Triphosphate; NHANES, National Health and Nutrition Examination Survey; KNN, K-nearest neighbors; LC-MS, liquid chromatography-mass spectrometry; QC, quality control; PCA, Principal Component Analysis; PLS-DA, Partial Least Squares Discriminant Analysis; SIRI, systemic inflammation response index; DIO1, Iodothyronine Deiodinase 1.

**Figure 4:**
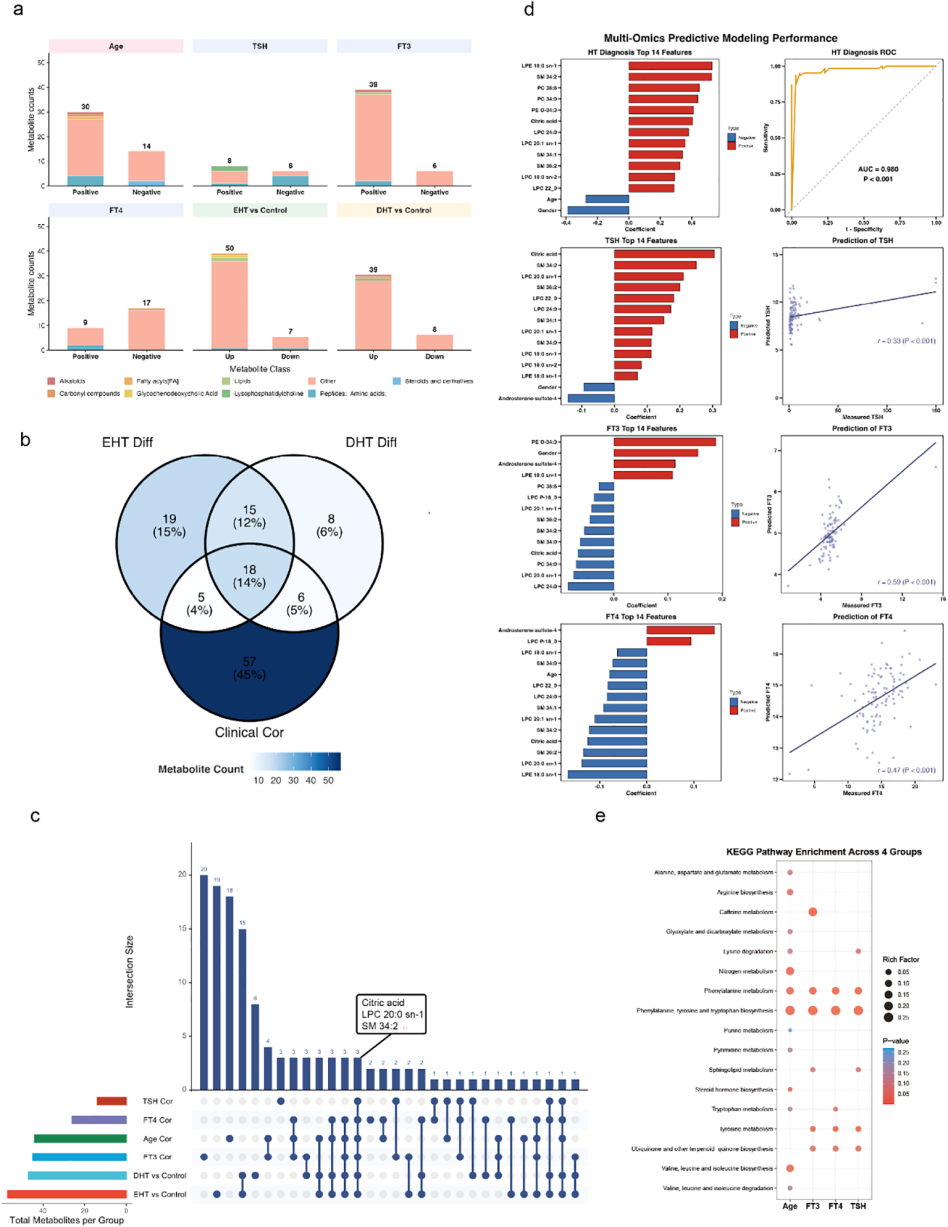
Screening, multidimensional intersection analysis, predictive modeling, and core marker identification for HT-related clinically associated metabolites. **(a)** Classification statistics for metabolites associated with age and thyroid function indicators (FT3, FT4, and TSH), as well as differential metabolites in EHT and DHT relative to CON. Age-related metabolites included 30 positive and 14 negative correlations; TSH-related metabolites included 8 positive and 6 negative correlations; FT3-related metabolites included 39 positive and 6 negative correlations; FT4-related metabolites included 9 positive and 17 negative correlations. Compared with CON, 50 metabolites were upregulated and 7 downregulated in EHT, and 39 were upregulated and 8 downregulated in DHT. **(b)** Venn intersection analysis of clinically associated metabolites and differential metabolites. Intersections among clinically associated metabolites, EHT differential metabolites, and DHT differential metabolites yielded 18 candidate metabolites. **(c)** UpSet plot showing intersections across six metabolite sets: age-related, FT3-related, FT4-related, TSH-related, EHT differential, and DHT differential metabolites. Only three metabolites were shared by all six sets. **(d)** HT diagnostic model and FT3, FT4, and TSH prediction models constructed from the 18 candidate metabolites and their performance. The HT diagnostic model had an ROC AUC of 0.980 (P <0.001); predicted values for TSH, FT3, and FT4 were significantly correlated with measured values. **(e)** KEGG pathway enrichment analysis of metabolites associated with age and thyroid function indicators.

Metabolite correlations with age and thyroid function indicators (FT3, FT4, and TSH) were first evaluated, and differential metabolic features of EHT and DHT relative to CON were compared. A total of 44 age-related metabolites, 14 TSH-related metabolites, 45 FT3-related metabolites, and 26 FT4-related metabolites were identified. Differential analyses showed that compared with CON, 57 differential metabolites were identified in EHT, including 50 upregulated and 7 downregulated metabolites; 47 differential metabolites were identified in DHT, including 39 upregulated and 8 downregulated metabolites (**Figure 4a**; **Supplementary Tables 8**, **S9**). These results indicate that overt metabolic profile changes are already present in HT patients even at the euthyroid stage.

KEGG pathway enrichment analyses were then performed for metabolites associated with age, FT3, FT4, and TSH. The related metabolites were mainly enriched in amino acid and lipid metabolism pathways, including phenylalanine metabolism, tyrosine metabolism, tryptophan metabolism, arginine biosynthesis, branched-chain amino acid metabolism, and sphingolipid metabolism (**Figure 4e**; **Supplementary Tables 11**-**S16**). These results suggest that HT-related metabolic abnormalities may primarily involve amino acid metabolic remodeling and lipid metabolic disturbance.

Next, clinical phenotype-related metabolite sets were intersected with EHT and DHT differential metabolites. Venn analysis identified 18 metabolites that simultaneously met the criteria of being significantly associated with clinical indicators and significantly changed in HT patients, suggesting that these metabolites may represent key candidate molecules linking clinical phenotypes and HT disease status (**Figure 4b**; **Supplementary Table 10**). UpSet analysis of six metabolite sets—age-related, FT3-related, FT4-related, TSH-related, EHT differential, and DHT differential metabolites—showed that only three metabolites were shared by all six sets: citric acid, LPC 20:0 sn-1, and SM 34:2. This finding suggests that although HT-related metabolic alterations are heterogeneous, a stable common metabolic basis may exist (**Figure 4c**).

Based on these 18 candidate metabolites, HT diagnostic models and FT3, FT4, and TSH prediction models were further constructed. The HT diagnostic model showed high discriminative ability, with an ROC AUC of 0.980 (P <0.001). For continuous-variable prediction, predicted TSH, FT3, and FT4 values were significantly positively correlated with measured values. The FT3 prediction model showed the strongest correlation (r = 0.59, P <0.001), followed by FT4 (r = 0.47, P <0.001) and TSH (r = 0.33, P <0.001) (**Figure 4d**). These results suggest that the 18 candidate metabolites not only have good ability to distinguish HT but also partly reflect thyroid functional status.

**Figure 5:**
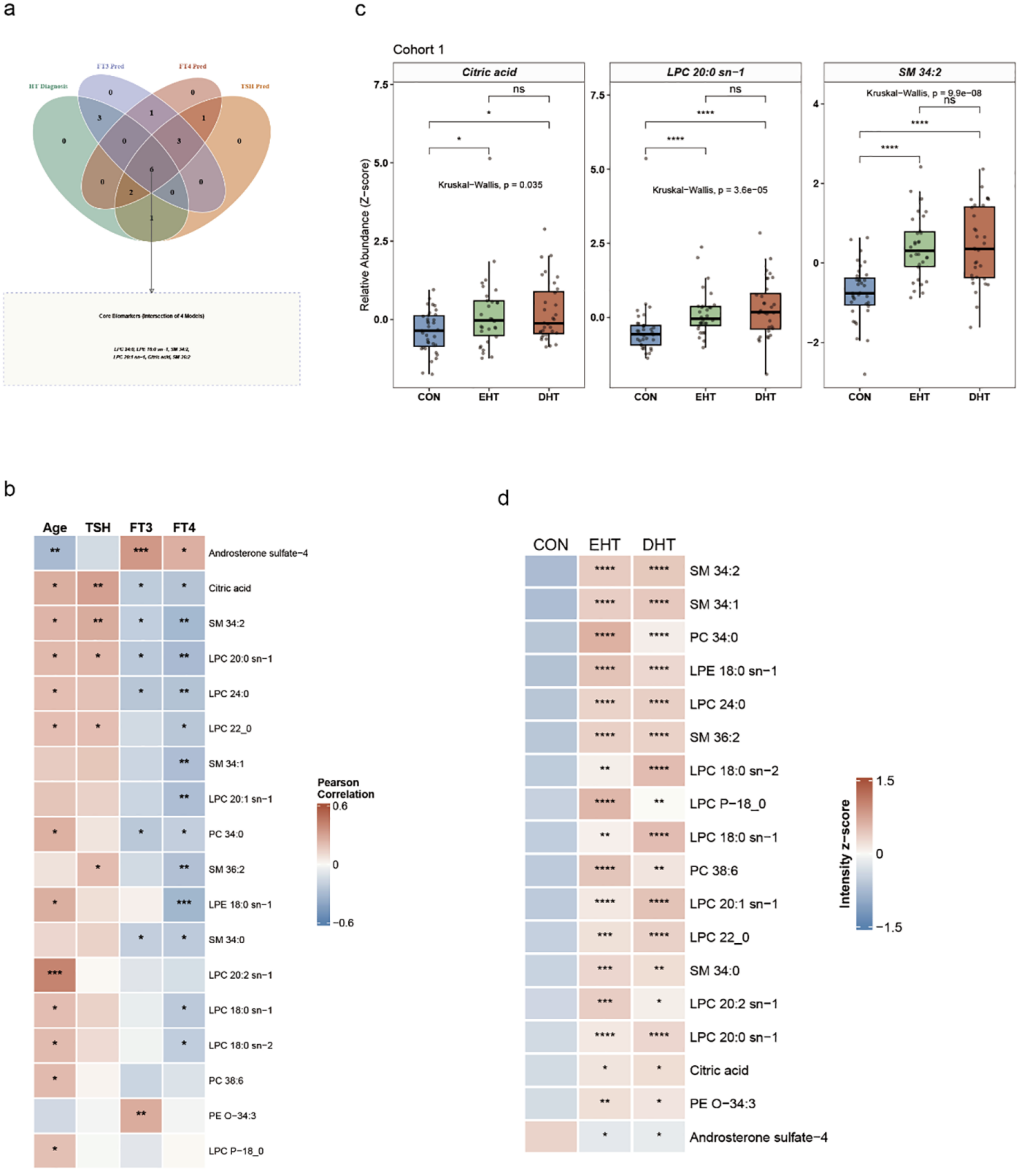
Identification of core markers in HT. **(a)** Venn plot of key features from four models. The HT diagnostic model and FT3, FT4, and TSH prediction models shared six intersecting metabolites: LPC 24:0, LPE 18:0 sn-1, SM 34:2, LPC 20:1 sn-1, citric acid, and SM 36:2. **(b)** Correlation heatmap between the 18 candidate metabolites and age, FT3, FT4, and TSH. **(c)** After integrating clinical correlation directions, between-group expression trends, and intersection analyses, three core metabolites were selected: citric acid, LPC 20:0 sn-1, and SM 34:2. Boxplots show their expression differences among groups in the discovery cohort. **(d)** Heatmap of expression levels of the 18 candidate metabolites across CON, EHT, and DHT groups.

To further identify more stable core markers, key high-contribution features from the four models were intersected. Six recurrent metabolites were obtained, including LPC 24:0, LPE 18:0 sn-1, SM 34:2, LPC 20:1 sn-1, citric acid, and SM 36:2 (**Figure 5a**). Correlation and between-group distribution analyses showed that most of these metabolites tended to be positively correlated with age and TSH but negatively correlated with FT3 and FT4. Their levels were also higher in EHT and DHT than in CON, with overall changes more evident in DHT (**Figures 5b, d**). This suggests that these metabolic abnormalities are present not only in HT patients with thyroid dysfunction but also in euthyroid HT patients.

After integrating clinical relevance, intergroup trends, multi−set intersections and model contributions, we prioritized three core candidate metabolites: citric acid, LPC 20:0 (sn−1) and SM 34:2.

We further reviewed the annotation confidence of these three metabolites. According to the LC−MS annotation report, citric acid was assigned a Level 2 annotation based on detection in negative ion mode with RT = 3.088 min, calculated m/z = 191.01987, molecular formula C_6_H_8_O_7_, mass error = 1.247 ppm, a database matching score of 88.04, and information from the BMDB/HMDB/KEGG databases. LPC 20:0 (sn−1) and SM 34:2 were retained as putatively annotated lipid metabolites based on the available LC−MS annotation records.

Boxplot analyses showed significant differences among CON, EHT, and DHT groups for all three metabolites: citric acid showed a statistically significant group difference (Kruskal-Wallis P = 0.035), LPC 20:0 sn-1 showed a stronger difference (P = 3.6 x 10^-5^), and SM 34:2 showed the strongest discriminatory ability (P = 9.9 x 10^-8^) (**Figure 5c**). Citric acid was also the metabolite with the highest contribution in the metabolic age model. Overall, levels of these metabolites were higher in EHT and DHT than in CON, whereas EHT and DHT did not differ significantly. These results suggest that citric acid, LPC 20:0 sn-1, and SM 34:2 may represent stable metabolic abnormalities appearing early in HT and may be key metabolites through which HT influences aging.

## 4 Discussion

This study systematically evaluated HT-related pro-aging phenotypes at three levels: biological age, metabolic age, and metabolomics. First, PhenoAge and its acceleration were elevated in HT patients in discovery cohorts, and directionally consistent external validation was obtained in the NHANES cohort using KDM biological age and PhenoAge. Second, at the metabolomics level, both EHT and DHT subgroups showed metabolic age acceleration, which was most pronounced in DHT. Finally, through metabolomics and machine learning screening, we identified core metabolites with aging relevance, HT diagnostic value, and disease progression implications, among which citric acid had the strongest integrative relevance. Overall, these results support that HT is not merely a thyroid-localized immune disease but a systemic disease state associated with a whole-body pro-aging phenotype (17).

This finding has clear biological plausibility. Chronic low-grade inflammation associated with aging (inflammaging) is considered an important shared mechanism for multiple age-related diseases, while the core pathological process of HT is also persistent immune activation, autoantibody production, and formation of a chronic inflammatory microenvironment (18). Long-term immune stress caused by HT may therefore interact with inflammaging at the mechanistic level and ultimately manifest clinically as increased biological age and age acceleration. Thyroid hormones also widely regulate energy metabolism, mitochondrial function, lipid metabolism, and muscle homeostasis, and their levels have bidirectional relationships with the aging process itself (19–22). The consistent findings across different cohorts and algorithms suggest that the HT-related pro-aging phenotype is unlikely to be an incidental result of a single indicator or cohort.

Autoimmune-driven chronic low−grade inflammation can regulate hepatic DIO1 via the NF−κB pathway and inhibit the conversion of T4 to active FT3, representing an important trigger for subclinical metabolic ageing in euthyroid patients with Hashimoto’s thyroiditis. Deiodinases are regulated by diverse inflammatory signals and can independently modulate thyroid hormone activation at the tissue level, a process that does not depend on peripheral thyroid function indices (23). A large−scale NHANES−based clinical study showed that an elevated systemic inflammation response index (SIRI) is significantly associated with increased levels of FT4, TT4 and TPOAb, providing population−level evidence that inflammation disrupts thyroid endocrine homeostasis (24). A recent experimental study found that a combination of taurine, resveratrol, retinol and oleic acid (a multi−nutrient formulation) restored hepatic DIO1 expression under inflammatory conditions, preserving peripheral thyroid hormone conversion and maintaining metabolic stability (25) On the basis of these findings, we hypothesize that nutritional interventions targeting the inflammation–DIO1 axis could ameliorate Hashimoto−related metabolic ageing, offering potential intervention strategies for the metabolite alterations (25). identified in this study.

Metabolomics results further support the HT-metabolic remodeling-aging axis (26). Previous metabolomics studies in HT and autoimmune thyroid disease have indicated that abnormalities in lipid molecules such as LPC, PC, and SM are relatively stable disease features. In hypothyroid states, systemic energy metabolism, lipid metabolism, and amino acid metabolism may also undergo substantial reprogramming. Building on these findings, our study further proposes that some metabolites are not only associated with disease status but also simultaneously reflect aging degree and thyroid functional changes, making them more likely to serve as bridge molecules linking disease onset, disease progression, and aging phenotypes. LPC and SM series molecules repeatedly appeared in intersection analyses and heatmaps, suggesting that membrane lipid metabolism and inflammation-related signaling pathways may play important roles in continuous HT progression (27, 28).

Citric acid showed the most prominent integrative value in this study. On the one hand, it was the top driving metabolite in the random forest metabolic age model, suggesting a close relationship with age-related metabolic phenotypes. On the other hand, it was included in the HT diagnostic model, thyroid function prediction models, and final core metabolite set, indicating that it is related not only to disease presence but also to disease progression. As a core intermediate of the TCA cycle, citric acid serves as a hub in immune cell activation, lipid synthesis, oxidative stress, and inflammatory mediator production (29–31). Existing studies show that intracellular citrate and ATP-citrate lyase-related pathways can support macrophage inflammatory responses and are linked to metabolic reprogramming in cellular senescence and age-related diseases (32, 33). Given the observed pattern in which citric acid was positively correlated with age and TSH but negatively correlated with FT3 and FT4, we speculate that citric acid may represent a metabolic signal integrating energy metabolic dysfunction, immune-inflammatory activation, and a tendency toward hypothyroidism. In other words, citric acid may be a key node worthy of further mechanistic validation in the HT pro-aging phenotype.

Clinically, the significance of this study is not merely to suggest that HT patients look older, but to indicate a systemic risk that may not be fully captured by conventional thyroid function testing (34–36). The EHT group already showed age acceleration and metabolic abnormalities, meaning that even when thyroid function remains within the normal range, patients may already exhibit systemic pro-aging changes. Future risk assessment in HT should therefore not be limited to TSH, FT3, FT4, and antibody levels, but should also consider integrating biological age, metabolic age, and key metabolites into comprehensive stratified management frameworks (37, 38). If subsequent studies further validate the reproducibility and mechanistic role of citric acid and related metabolic pathways, it may become a new metabolic marker for auxiliary diagnosis, stratified monitoring, and progression warning.

## 5 Limitations

First, this study was primarily observational, and the metabolomics component of the discovery cohort was mainly cross-sectional; therefore, the relationship between HT and pro-aging phenotypes should currently be interpreted as a significant association rather than strict causality. Although external validation was performed in NHANES, longer-term prospective follow-up studies are still needed to determine whether HT truly accelerates subsequent aging outcomes and multimorbidity risk.

Second, the indicator systems differed between discovery and validation cohorts. Discovery cohorts focused on PhenoAge, metabolic age, and metabolomics features, whereas the validation cohort focused on KDM biological age and PhenoAge. This design allows cross-support from different perspectives, but it also means that the metabolomics model and core metabolites still lack direct replication in an independent external metabolomics cohort.

Third, the roles of core metabolites, especially citric acid, LPC 20:0 sn-1, and SM 34:2, currently remain supported by statistical and bioinformatics evidence; cellular, animal, or functional experiments are still lacking to clarify their specific mechanisms in HT immune inflammation, hypothyroid progression, and aging remodeling.

Fourth, although the random forest-based metabolomic age model captured age-related metabolic changes in healthy controls, its apparent performance in the training set was notably higher than its cross−validated performance, suggesting a risk of overfitting. This limitation may be attributable to the limited sample size of healthy controls and the high dimensionality of the LC−MS metabolomics data. Future optimization and validation of the metabolomic age model will require larger healthy reference populations and independent external metabolomic cohorts.

Therefore, this study should be viewed as an integrative work proposing an HT-related pro-aging phenotype and candidate metabolic pathways, providing a basis for future large-sample, multicenter, longitudinal, and mechanistic studies.

## 6 Conclusions

Hashimoto’s thyroiditis is closely associated with increased biological age, metabolic age acceleration, and pro-aging metabolic remodeling. Based on metabolomics integrative analysis, citric acid was identified as a key metabolic molecule with aging relevance, HT diagnostic value, and implications for disease progression. These findings suggest that HT may not only be a thyroid-localized autoimmune disease but also a systemic disease associated with whole-body pro-aging status.

## Data Availability

The original contributions presented in the study are included in the article/**Supplementary Material**. Further inquiries can be directed to the corresponding authors.

## Supplementary material

Supplementary Figure 1 Sex-stratified comparisons of biological age, age acceleration, and accelerated proportions between patients with HT and healthy controls in discovery cohorts 1 and 2. **(a-c)** Distributions of biological age and chronological age: KDM age in discovery cohort 1 **(a)**, PhenoAge in discovery cohort 1 (b), and KDM age in discovery cohort 2 **(d-h)** Sex-stratified comparisons between healthy controls and HT patients in discovery cohort 1 for KDM biological age **(d)**, KDM age acceleration **(e)**, PhenoAge **(f)**, PhenoAge acceleration **(g)**, and the proportion with KDM age or PhenoAge acceleration **(i-j)** Sex-stratified comparisons between healthy controls and HT patients in discovery cohort 2 for KDM biological age **(i)** and KDM age acceleration **(j)**.

Supplementary Figure 2 Biological age, age acceleration, and accelerated proportions in male HT patients and healthy controls in the NHANES cohort. **(a-f)** Comparisons between male healthy controls and male HT patients in NHANES for KDM biological age **(a)**, KDM age acceleration **(b)**, PhenoAge **(c)**, PhenoAge acceleration **(d)**, the proportion with KDM age acceleration **(e)**, and the proportion with PhenoAge acceleration **(g-i)** Comparisons among male healthy controls and male HT patients at different stages in NHANES for KDM biological age **(g)**, KDM age acceleration **(h)**, and PhenoAge **(i)**. P <0.05 was considered statistically significant.

Supplementary Figure 3 Biological age, age acceleration, and accelerated proportions in female HT patients and healthy controls in the NHANES cohort. **(a-f)** Comparisons between female healthy controls and female HT patients in NHANES for KDM biological age **(a)**, KDM age acceleration **(b)**, PhenoAge **(c)**, PhenoAge acceleration **(d)**, the proportion with KDM age acceleration **(e)**, and the proportion with PhenoAge acceleration **(g-i)** Comparisons among female healthy controls and female HT patients at different stages in NHANES for KDM biological age **(g)**, KDM age acceleration **(h)**, and PhenoAge **(i)**. P <0.05 was considered statistically significant.

Supplementary Figure 4 Sex-stratified metabolic age features and metabolic age acceleration analyses. **(a)** Test-set and training-set performance of the random forest metabolic age model validated by five-fold cross-validation. **(b)** Sex-stratified correlations between chronological age and predicted metabolic age. Scatterplots show the relationship between chronological age and random forest-predicted metabolic age in females (left) and males (right). Each point represents one participant, colored by CON, EHT, or DHT. Black lines indicate fitted trends. **(c)** Sex-stratified comparisons of metabolic age across groups. Distributions of metabolic age were compared among CON, EHT, and DHT in females (left) and males (right). Each point represents one participant; violin plot width indicates density; overlaid boxplots show medians and interquartile ranges. **(d)** Sex-stratified differences in MAA across groups. MAA levels were compared among CON, EHT, and DHT in females (left) and males (right). Gray points indicate individual values, and colored bars with error bars indicate group summary statistics. **(e)** Sex-stratified proportions of metabolic age acceleration across groups. Stacked bar charts show accelerated and non-accelerated metabolic age status among CON, EHT, and DHT in females (left) and males (right). Red indicates acceleration and blue indicates non-acceleration.

Supplementary Figure 5 PCA and PLS-DA of the HT metabolomics cohort. The left panel shows principal component analysis (PCA), and the right panel shows partial least squares-discriminant analysis (PLS-DA). Each point represents one participant, with colors indicating CON, EHT, and DHT; dashed ellipses indicate the overall clustering ranges of groups. PCA showed partial overlap among the three groups but suggested distributional differences, whereas PLS-DA further showed partial separation, suggesting metabolomic differences among study groups.
