# Supplementary material for: Metabolic dysregulation and biological age acceleration in Hashimoto’s thyroiditis: a cross-sectional study based on clinical biomarker aging indices and metabolomics
Source: Front Endocrinol (Lausanne). 2026 Jun 26;17:1874574. doi: 10.3389/fendo.2026.1874574 (PMC13350340; doi:10.3389/fendo.2026.1874574)
Supplement: Supplementary Figure 1 — Sex-stratified comparisons of biological age, age acceleration, and accelerated proportions between patients with HT and healthy controls in discovery cohorts 1 and 2. (a-c) Distributions of biological age and chronological age: KDM age in discovery cohort 1 (a), PhenoAge in discovery cohort 1 (b), and KDM age in discovery cohort 2 (d-h) Sex-stratified comparisons between healthy controls and HT patients in discovery cohort 1 for KDM biological age (d), KDM age acceleration (e), PhenoAge (f), PhenoAge acceleration (g), and the proportion with KDM age or PhenoAge acceleration (i-j) Sex-stratified comparisons between healthy controls and HT patients in discovery cohort 2 for KDM biological age (i) and KDM age acceleration (j). [file DataSheet1.docx]

**Supplementary Materials**

**Supplementary Material 1. Cohorts and overview of main analyses.**

| **Cohort** | **Group** | **Sample size** | **Main indicators** | **Analytical role** |
| --- | --- | --- | --- | --- |
| Discovery cohort 1 | Healthy controls vs HT | 249 vs 159 | KDM biological age; PhenoAge; age acceleration; thyroid-function and clinical indicators | primary discovery analysis |
| Discovery cohort 2 | Healthy controls vs HT | 27 vs 31 | KDM biological age; KDM age acceleration; thyroid-function and clinical indicators | local independent replication |
| NHANES 2007-2012 | Healthy controls vs HT；stratified by thyroid functional status | 2,483 vs 705; stratified analysis: EHT 480, subclinical hypothyroid HT 56, overt hypothyroid HT 45 | KDM biological age; PhenoAge; socioeconomic and lifestyle covariates | external population validation |
| metabolomicsCohort | CON、EHT、DHT | 77、48、36 | metabolomics; metabolic age; thyroid function | metabolic age and candidate metabolite analyses |

**Supplementary Material 2. NHANES participant selection flow.**

**
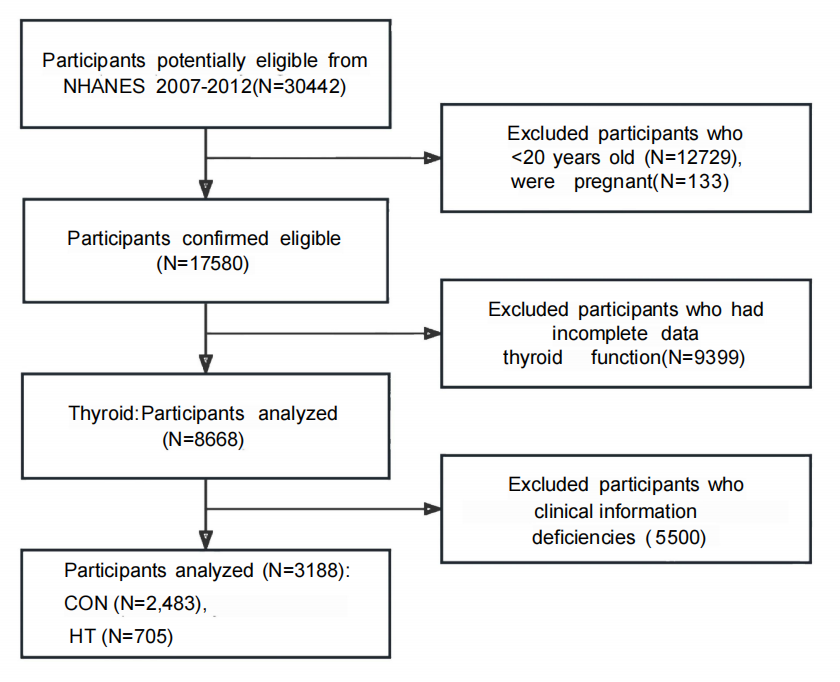
**

**Supplementary Material 3. Supplementary notes on biological age calculation.**

| **Cohort** | **PhenoAge** | **KDM Age** | **Missing-data handling** | **Acceleration threshold** |
| --- | --- | --- | --- | --- |
| Discovery cohort 1 | Calculated using the standard PhenoAge formula | sample-specific modified KDM | No cross-cohort imputation; outcomes were set to missing if model variables were missing | acc > 0 |
| Discovery cohort 2 | Not calculated | sample-specific modified KDM | No cross-cohort imputation; outcomes were set to missing if model variables were missing | acc > 0 |
| Validation Cohort NHANES | Calculated using the BioAge package | Calculated using the BioAge package | KNN, k=5; outcome variables were not imputed | acc > 0 |

**References**

1 Klemera P, Doubal S. A new approach to the concept and computation of biological age. Mechanisms of Ageing and Development. 2006.

2 Liu Z, Kuo PL, Horvath S, Crimmins E, Ferrucci L, Levine M. A new aging measure captures morbidity and mortality risk across diverse subpopulations from NHANES IV: a cohort study. PLoS Medicine. 2018.

3 Kwon D, Belsky DW. A toolkit for quantification of biological age from blood chemistry and organ function test data. eLife. 2021.

**Supplementary Material 4 . Sensitivity analysis of biological age, age acceleration, and accelerated proportions in the NHANES cohort after excluding participants using thyroid-related medications.**

(a-f) Comparisons between healthy controls and HT patients after excluding participants using thyroid-related medications for KDM biological age (a), KDM age acceleration (b), PhenoAge (c), PhenoAge acceleration (d), the proportion with KDM age acceleration (e), and the proportion with PhenoAge acceleration (f).

(g-i) Comparisons among healthy controls and HT patients at different thyroid functional stages after excluding participants using thyroid-related medications for KDM biological age (g), KDM age acceleration (h), and PhenoAge (i). P <0.05 was considered statistically significant.


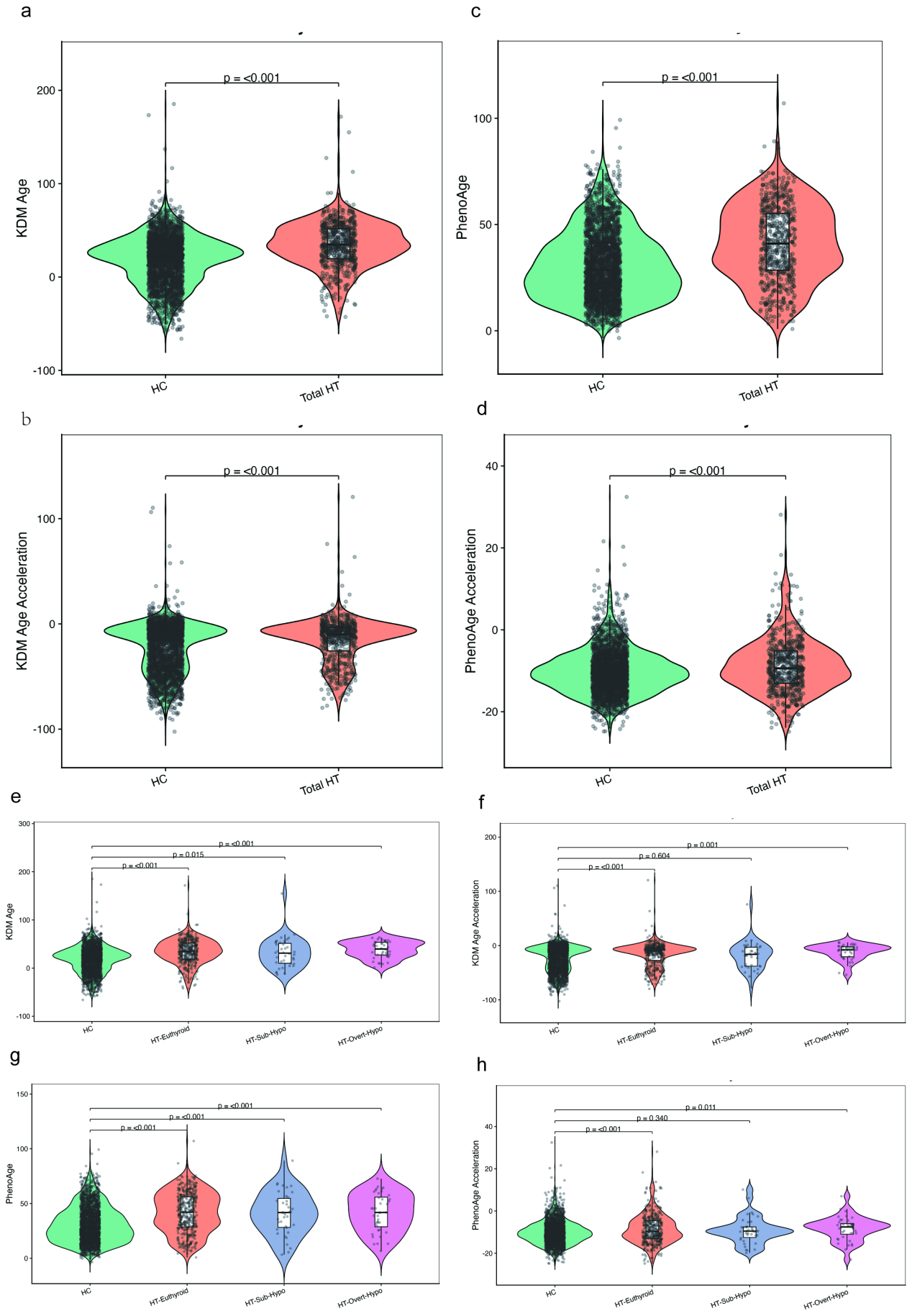


**(added) Supplementary Material 5 . Sensitivity analysis of biological age, age acceleration, and accelerated proportions in male participants in the NHANES cohort after excluding participants using thyroid-related medications.**

(a-f) Comparisons between healthy controls and HT patients after excluding participants using thyroid-related medications for KDM biological age (a), KDM age acceleration (b), PhenoAge (c), PhenoAge acceleration (d), the proportion with KDM age acceleration (e), and the proportion with PhenoAge acceleration (f).

(g-i) Comparisons among healthy controls and HT patients at different thyroid functional stages after excluding participants using thyroid-related medications for KDM biological age (g), KDM age acceleration (h), and PhenoAge (i). P <0.05 was considered statistically significant.


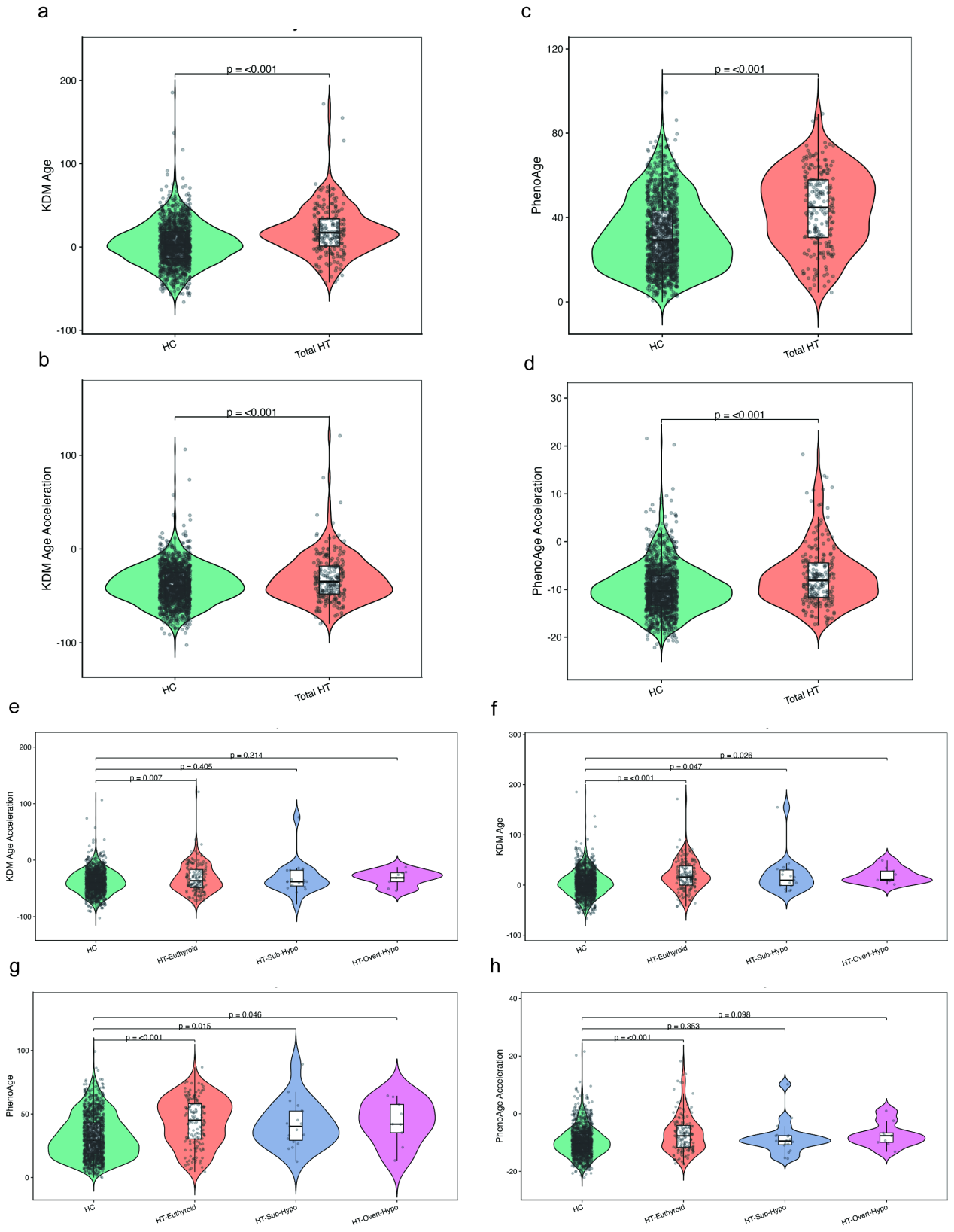


**(added) Supplementary Material 6. Sensitivity analysis of biological age, age acceleration, and accelerated proportions in female participants in the NHANES cohort after excluding participants using thyroid-related medications.**

(a-f) Comparisons between healthy controls and HT patients after excluding participants using thyroid-related medications for KDM biological age (a), KDM age acceleration (b), PhenoAge (c), PhenoAge acceleration (d), the proportion with KDM age acceleration (e), and the proportion with PhenoAge acceleration (f).

(g-i) Comparisons among healthy controls and HT patients at different thyroid functional stages after excluding participants using thyroid-related medications for KDM biological age (g), KDM age acceleration (h), and PhenoAge (i). P <0.05 was considered statistically significant.


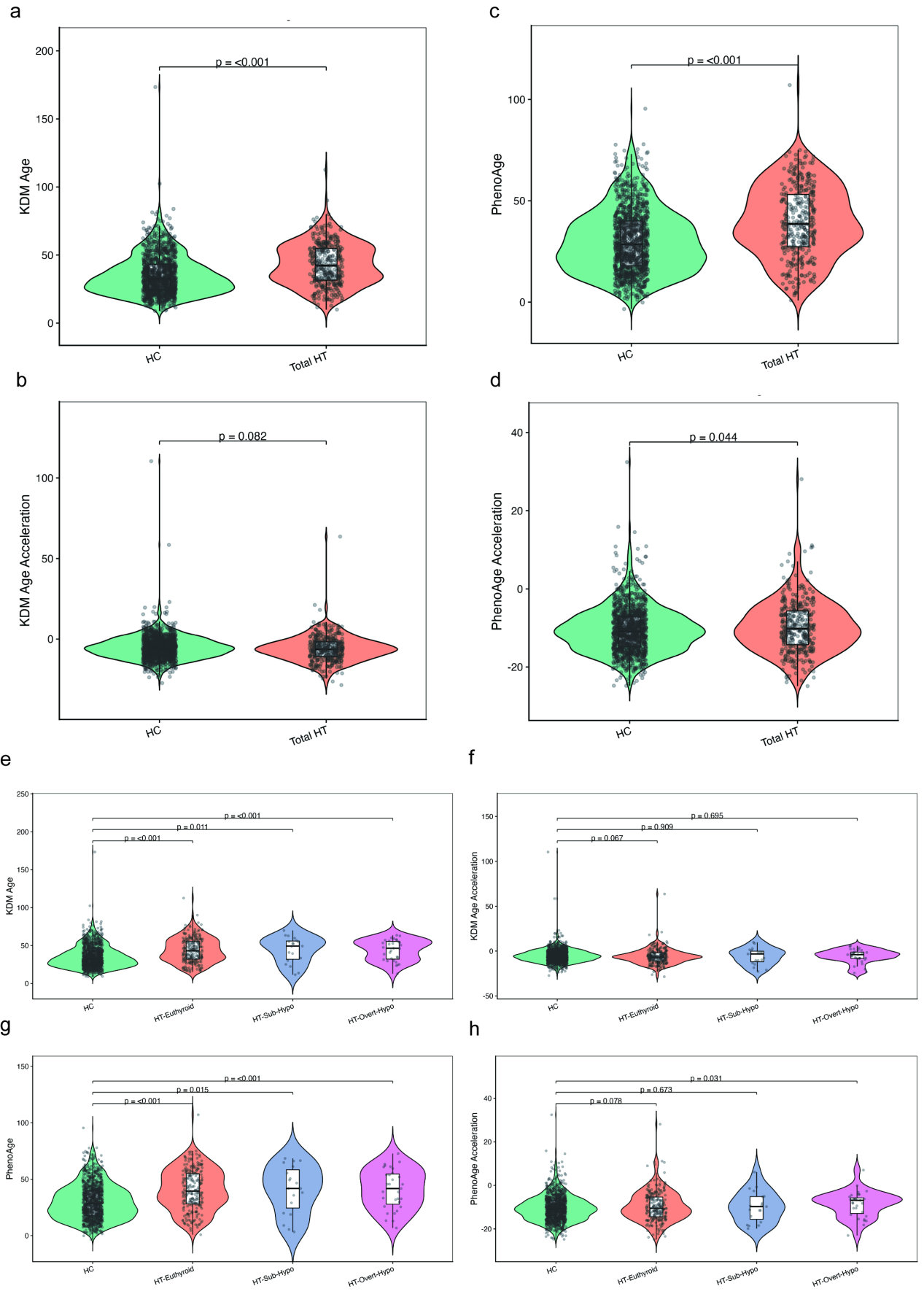


**(added) Supplementary Material 7. Baseline clinical characteristics and biological age indicators in the NHANES validation cohort after excluding participants using thyroid-related medications.**

| **Characteristic** | **Healthy Control**  **n=2426** | **Hashimoto's Thyroiditis n=576** | **p** |
| --- | --- | --- | --- |
| Age (years) | 39.00 [29.00, 51.00] | 50.00 [38.00, 62.00] | <0.001 |
| Gender (%) |  |  | <0.001 |
| Female | 1142 (47.1%) | 358 (62.2%) |  |
| Male | 1284 (52.9%) | 218 (37.8%) |  |
| Race (%) |  |  | 0.003 |
| Mexican American | 421 (17.4%) | 131 (22.7%) |  |
| Non-Hispanic Black | 404 (16.7%) | 70 (12.2%) |  |
| Non-Hispanic White | 1229 (50.7%) | 276 (47.9%) |  |
| Other | 104 (4.3%) | 23 (4.0%) |  |
| Other Hispanic | 268 (11.0%) | 76 (13.2%) |  |
| PIR level (%) |  |  | 0.275 |
| Below poverty | 507 (20.9%) | 111 (19.3%) |  |
| High income | 679 (28.0%) | 165 (28.6%) |  |
| Low income | 599 (24.7%) | 128 (22.2%) |  |
| Middle income | 641 (26.4%) | 172 (29.9%) |  |
| Education level (%) |  |  | 0.057 |
| college or above | 1268 (52.3%) | 281 (48.8%) |  |
| high school or equivalent | 579 (23.9%) | 130 (22.6%) |  |
| less than high school | 579 (23.9%) | 165 (28.6%) |  |
| Drinking (%) |  |  | 0.064 |
| heavy drinker | 263 (10.8%) | 52 (9.0%) |  |
| low to moderate drinker | 664 (27.4%) | 184 (31.9%) |  |
| non-drinker | 1499 (61.8%) | 340 (59.0%) |  |
| Smoking status (%) |  |  | 0.004 |
| Current smoker | 613 (25.3%) | 111 (19.3%) |  |
| Former smoker | 501 (20.7%) | 143 (24.8%) |  |
| Never smoker | 1312 (54.1%) | 322 (55.9%) |  |
| Physical activity (%) |  |  | 0.064 |
| High physical activity | 1826 (75.3%) | 407 (70.7%) |  |
| Low physical activity | 320 (13.2%) | 94 (16.3%) |  |
| Middle physical activity | 280 (11.5%) | 75 (13.0%) |  |
| FT3 (pg/ml) | 3.20 [3.00, 3.50] | 3.10 [2.90, 3.40] | <0.001 |
| TT3 (ng/dL) | 114.00 [101.25, 128.00] | 111.00 [98.00, 127.00] | 0.009 |
| FT4 (pmol/L) | 10.30 [9.00, 10.60] | 9.30 [9.00, 10.62] | <0.001 |
| TT4 (ug/dL) | 7.60 [6.80, 8.60] | 7.60 [6.70, 8.70] | 0.637 |
| TSH (mIU/L) | 1.46 [1.02, 2.08] | 2.12 [1.30, 3.30] | <0.001 |
| TPOAB (IU/mL) | 0.60 [0.30, 1.10] | 36.95 [10.40, 176.80] | <0.001 |
| TGAB (IU/mL) | 0.60 [0.60, 0.60] | 3.05 [0.60, 16.52] | <0.001 |
| Albumin (g/L) | 43.00 [41.00, 45.00] | 42.00 [40.00, 44.00] | <0.001 |
| ALP (IU/L) | 64.00 [53.00, 77.00] | 67.00 [55.00, 80.00] | <0.001 |
| BUN (mg/dl) | 11.00 [9.00, 14.00] | 12.00 [10.00, 15.00] | <0.001 |
| Serum Creatinine(μmol/L) | 72.49 [63.65, 85.75] | 72.49 [60.11, 81.55] | 0.007 |
| CRP (mg/dL) | 0.14 [0.06, 0.33] | 0.18 [0.08, 0.39] | <0.001 |
| HbA1c (%) | 5.30 [5.10, 5.50] | 5.50 [5.30, 5.90] | <0.001 |
| Total cholesterol (mg/dl) | 192.00 [167.00, 219.00] | 197.00 [171.00, 225.00] | 0.01 |
| Glucose (mmol) | 4.83 [4.50, 5.11] | 5.16 [4.77, 5.72] | <0.001 |
| Lymphocyte percent (%) | 30.50 [25.80, 35.68] | 30.65 [26.08, 35.60] | 0.742 |
| MCV (fl) | 89.10 [86.30, 92.00] | 89.40 [86.30, 92.30] | 0.7 |
| RDW (%) | 12.50 [12.10, 12.90] | 12.60 [12.20, 13.22] | <0.001 |
| WBC (x1000 cells) | 6.90 [5.60, 8.20] | 6.90 [5.70, 8.20] | 0.453 |
| FEV1 (mL) | 3284.50 [2765.25, 3928.75] | 2845.00 [2286.00, 3480.50] | <0.001 |
| SBP (mmHg) | 118.00 [110.00, 128.00] | 122.00 [112.00, 134.00] | <0.001 |
| KDM Age（years） | 22.10 [0.80, 35.44] | 34.71 [19.73, 51.60] | <0.001 |
| KDMAge Acccceleration（years） | -14.94 [-40.58, -5.30] | -9.99 [-25.69, -3.29] | <0.001 |
| PhenoAge (years) | 28.68 [18.32, 41.50] | 41.05 [28.55, 55.23] | <0.001 |
| PhenoAge Acccceleration (years) | -10.54 [-13.87, -7.05] | -9.40 [-13.03, -5.17] | <0.001 |
| KDMAge Accelerated (%) | 279/2426 (11.5%) | 85/576 (14.8%) | 0.031 |
| PhenoAge Accelerated (%) | 99/2426 (4.1%) | 49/576 (8.5%) | <0.001 |

**(added) Supplementary Material 8. Baseline clinical characteristics and biological age indicators according to HT stage in the NHANES validation cohort after excluding participants using thyroid-related medications.**

| **Characteristic** | **Healthy Control n=2426** | **Hashimoto's Thyroiditis Euthyroidism n=401** | **Hashimoto's Thyroiditis Subclinical Hypothyridism n=38** | **Hashimoto's Thyroiditis Overt Hypothyridism n=40** | **p** |
| --- | --- | --- | --- | --- | --- |
| Age (years) | 39.00[29.00,51.00] | 51.00[37.00,64.00] | 52.00[38.25,60.75] | 53.50[37.50,64.25] | <0.001 |
| Gender, No. (%) |  |  |  |  | <0.001 |
| Female | 1142 (47.1%) | 242 (60.3%) | 19 (50.0%) | 31 (77.5%) |  |
| Male | 1284 (52.9%) | 159 (39.7%) | 19 (50.0%) | 9 (22.5%) |  |
| Race, No. (%) |  |  |  |  | 0.002 |
| Mexican American | 421 (17.4%) | 95 (23.7%) | 2 (5.3%) | 6 (15.0%) |  |
| Non-Hispanic Black | 404 (16.7%) | 57 (14.2%) | 1 (2.6%) | 6 (15.0%) |  |
| Non-Hispanic White | 1229 (50.7%) | 179 (44.6%) | 29 (76.3%) | 23 (57.5%) |  |
| Other | 104 (4.3%) | 21 (5.2%) | 0 (0.0%) | 0 (0.0%) |  |
| Other Hispanic | 268 (11.0%) | 49 (12.2%) | 6 (15.8%) | 5 (12.5%) |  |
| PIR level, No. (%) |  |  |  |  | 0.565 |
| Below poverty | 507 (20.9%) | 79 (19.7%) | 6 (15.8%) | 5 (12.5%) |  |
| High income | 679 (28.0%) | 112 (27.9%) | 15 (39.5%) | 11 (27.5%) |  |
| Low income | 599 (24.7%) | 97 (24.2%) | 7 (18.4%) | 8 (20.0%) |  |
| Middle income | 641 (26.4%) | 113 (28.2%) | 10 (26.3%) | 16 (40.0%) |  |
| Education level, No. (%) |  |  |  |  | 0.012 |
| college or above | 1268 (52.3%) | 184 (45.9%) | 21 (55.3%) | 26 (65.0%) |  |
| high school or equivalent | 579 (23.9%) | 88 (21.9%) | 8 (21.1%) | 8 (20.0%) |  |
| less than high school | 579 (23.9%) | 129 (32.2%) | 9 (23.7%) | 6 (15.0%) |  |
| Drinking, No. (%) |  |  |  |  | 0.063 |
| heavy drinker | 263 (10.8%) | 32 (8.0%) | 5 (13.2%) | 4 (10.0%) |  |
| low to moderate drinker | 664 (27.4%) | 117 (29.2%) | 12 (31.6%) | 19 (47.5%) |  |
| non-drinker | 1499 (61.8%) | 252 (62.8%) | 21 (55.3%) | 17 (42.5%) |  |
| Smoking status, No. (%) |  |  |  |  | 0.084 |
| Current smoker | 613 (25.3%) | 78 (19.5%) | 6 (15.8%) | 6 (15.0%) |  |
| Former smoker | 501 (20.7%) | 98 (24.4%) | 9 (23.7%) | 11 (27.5%) |  |
| Never smoker | 1312 (54.1%) | 225 (56.1%) | 23 (60.5%) | 23 (57.5%) |  |
| Physical activity, No. (%) |  |  |  |  | 0.208 |
| High physical activity | 1826 (75.3%) | 280 (69.8%) | 29 (76.3%) | 34 (85.0%) |  |
| Low physical activity | 320 (13.2%) | 68 (17.0%) | 4 (10.5%) | 4 (10.0%) |  |
| Middle physical activity | 280 (11.5%) | 53 (13.2%) | 5 (13.2%) | 2 (5.0%) |  |
| FT3 (pg/ml) | 3.12 [2.90, 3.40] | 3.20 [2.92, 3.40] | 2.90 [2.69, 3.20] | <0.001 | 3.12 [2.90, 3.40] |
| TT3 (ng/dL) | 111.00 [98.00, 128.00] | 109.00 [99.25, 135.50] | 103.00 [93.75, 113.25] | 0.002 | 111.00 [98.00, 128.00] |
| FT4 (pmol/L) | 10.30 [9.00, 11.60] | 10.30 [9.00, 10.30] | 7.70 [6.50, 7.70] | <0.001 | 10.30 [9.00, 11.60] |
| TT4 (ug/dL) | 7.70 [7.00, 8.90] | 7.45 [6.50, 8.70] | 5.95 [5.00, 6.60] | <0.001 | 7.70 [7.00, 8.90] |
| TSH (mIU/L) | 1.80 [1.21, 2.69] | 5.87 [5.20, 7.32] | 7.60 [5.39, 18.96] | <0.001 | 1.80 [1.21, 2.69] |
| TPOAB (IU/mL) | 26.70 [9.50, 142.40] | 158.50 [37.95, 539.73] | 241.65 [36.95, 398.08] | <0.001 | 26.70 [9.50, 142.40] |
| TGAB (IU/mL) | 3.00 [0.60, 15.30] | 4.45 [0.60, 23.47] | 8.75 [0.90, 34.58] | <0.001 | 3.00 [0.60, 15.30] |
| Albumin (g/L) | 43.00 [41.00, 44.00] | 42.00 [40.00, 44.00] | 43.00 [41.00, 45.00] | <0.001 | 43.00 [41.00, 44.00] |
| ALP (IU/L) | 67.00 [56.00, 81.00] | 70.50 [57.25, 77.75] | 61.00 [51.75, 68.50] | <0.001 | 67.00 [56.00, 81.00] |
| BUN (mg/dl) | 12.00 [10.00, 15.00] | 12.00 [11.00, 15.00] | 12.00 [10.75, 14.00] | 0.002 | 12.00 [10.00, 15.00] |
| Serum Creatinine(μmol/L) | 72.49 [60.11, 81.33] | 81.33 [70.50, 89.29] | 72.49 [63.43, 86.85] | 0.006 | 72.49 [60.11, 81.33] |
| CRP (mg/dL) | 0.18 [0.08, 0.40] | 0.15 [0.07, 0.40] | 0.16 [0.10, 0.41] | 0.003 | 0.18 [0.08, 0.40] |
| HbA1c (%) | 5.60 [5.30, 6.00] | 5.40 [5.23, 5.70] | 5.40 [5.20, 5.73] | <0.001 | 5.60 [5.30, 6.00] |
| Total cholesterol (mg/dl) | 192.00 [167.00, 219.00] | 196.00 [170.00, 220.00] | 199.00 [179.75, 226.00] | 204.50 [179.50, 242.50] | 0.042 |
| Glucose (mmol) | 4.83 [4.50, 5.11] | 5.16 [4.77, 5.77] | 5.02 [4.73, 5.49] | 5.02 [4.65, 5.61] | <0.001 |
| Lymphocyte percent (%) | 30.50 [25.80, 35.68] | 30.30 [25.50, 35.40] | 30.95 [26.38, 35.75] | 31.95 [28.43, 39.55] | 0.294 |
| MCV (fl) | 89.10 [86.30, 92.00] | 89.40 [85.90, 92.10] | 90.00 [86.82, 92.75] | 89.00 [86.55, 93.20] | 0.818 |
| RDW (%) | 12.50 [12.10, 12.90] | 12.60 [12.20, 13.30] | 12.40 [12.05, 12.97] | 12.90 [12.30, 13.85] | <0.001 |
| WBC (x1000 cells) | 6.90 [5.60, 8.20] | 7.00 [5.70, 8.30] | 6.35 [5.32, 7.97] | 7.25 [5.75, 8.30] | 0.304 |
| FEV1 (mL) | 3284.50 [2765.25, 3928.75] | 2860.00 [2326.00, 3484.00] | 3097.50 [2149.50, 3626.50] | 2646.50 [1981.75, 3312.75] | <0.001 |
| SBP (mmHg) | 118.00 [110.00, 128.00] | 122.00 [112.00, 134.00] | 124.00 [112.50, 136.50] | 119.00 [111.50, 132.00] | <0.001 |
| KDM Age（years） | 22.10 [0.80, 35.44] | 35.14 [18.21, 51.69] | 31.11 [10.05, 51.50] | 39.69 [27.23, 53.56] | <0.001 |
| KDMAge Acccceleration（years） | -14.94 [-40.58, -5.30] | -9.75 [-27.29, -4.23] | -16.12 [-37.88, -3.11] | -7.88 [-20.48, -2.07] | <0.001 |
| PhenoAge (years) | 28.68 [18.32, 41.50] | 42.11 [28.59, 56.08] | 41.79 [28.26, 54.73] | 41.88 [28.79, 55.90] | <0.001 |
| PhenoAge Acccceleration (years) | -10.54 [-13.87, -7.05] | -9.61 [-13.02, -4.57] | -9.52 [-12.52, -7.51] | -7.54 [-10.99, -5.95] | <0.001 |
| KDMAge Accelerated (%) | 279/2426 (11.5%) | 57/401 (14.2%) | 6/38 (15.8%) | 7/40 (17.5%) | 0.2 |
| PhenoAge Accelerated (%) | 99/2426 (4.1%) | 38/401 (9.5%) | 2/38 (5.3%) | 2/40 (5.0%) | <0.001 |

**(added) Supplementary Material 9. No crp KDMAge and PhenoAge, age acceleration, and accelerated proportions in patients with HT and healthy controls in discovery cohorts 1 .**(a-b) Comparisons between healthy controls and HT patients in discovery cohort 1 for KDM biological age (KdmAge) (a), KDM age acceleration (b), (c-d) Comparisons between healthy controls and HT patients in discovery cohort 1 for KDM biological age (KdmAge) (c), KDM age acceleration (d), (e)proportions of participants with KDM age or PhenoAge acceleration in cohorts 1. **
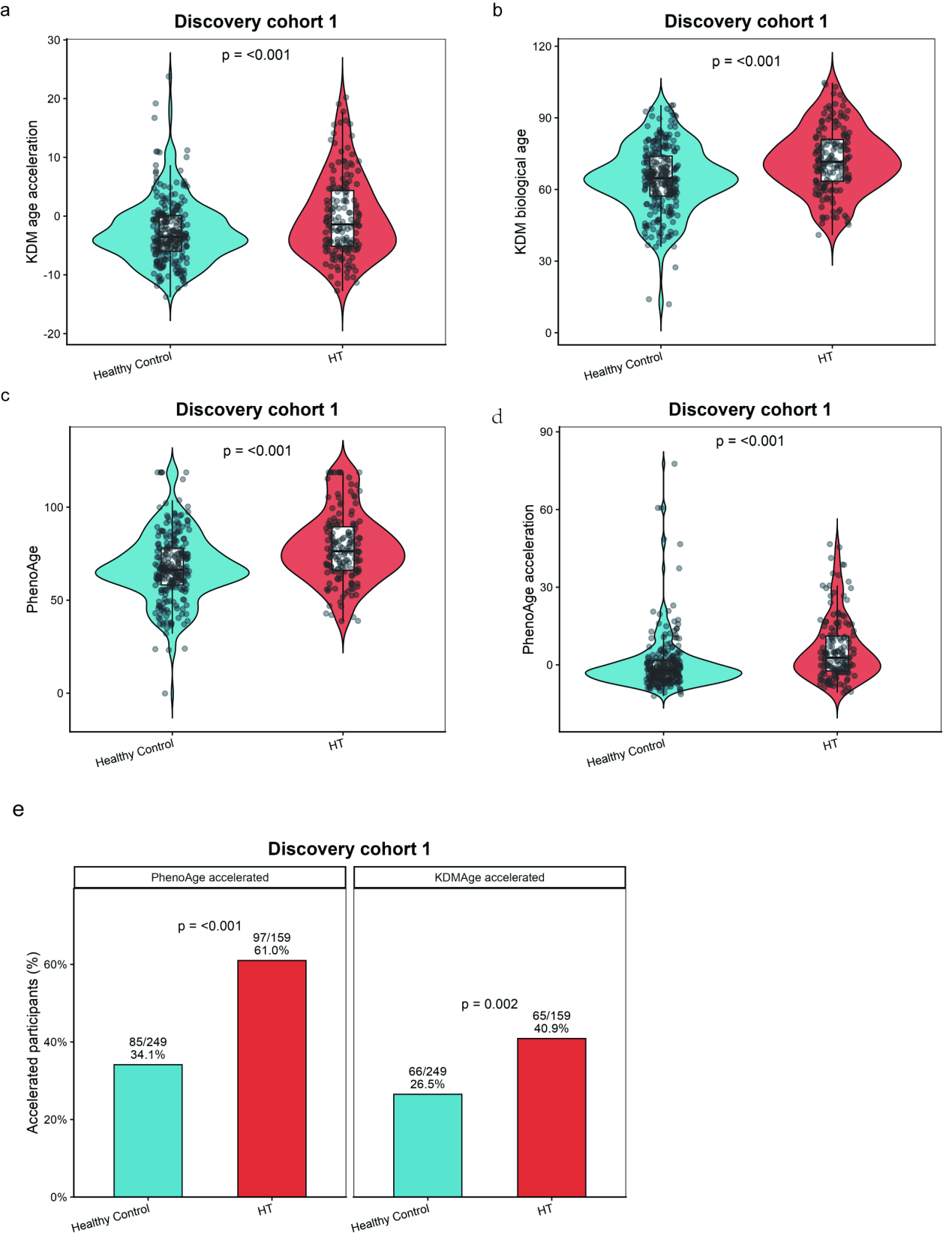
**

**(added) Supplementary Material 10. The regression analysis of the association between Hashimoto’s thyroiditis and no crp KDMAge and PhenoAge in Cohort 1**

| Cohort | Outcome | Outcome type | Model | Comparison | Effect measure | Estimate (95% CI) | P value |
| --- | --- | --- | --- | --- | --- | --- | --- |
| Cohort 1 | PhenoAge, y | Continuous | Adjusted for Age + Gender | HT vs healthy control | β | 6.55 (4.25 to 8.85) | <0.001 |
| Cohort 1 | KDM biological age, y | Continuous | Adjusted for Age + Gender | HT vs healthy control | β | 3.35 (2.12 to 4.57) | <0.001 |
| Cohort 1 | PhenoAge acceleration, y | Continuous | Adjusted for Age + Gender | HT vs healthy control | β | 6.55 (4.25 to 8.85) | <0.001 |
| Cohort 1 | KDM age acceleration, y | Continuous | Adjusted for Age + Gender | HT vs healthy control | β | 3.35 (2.12 to 4.57) | <0.001 |
| Cohort 1 | PhenoAge accelerated | Binary | Adjusted for Age + Gender | HT vs healthy control | OR | 3.80 (2.40 to 6.04) | <0.001 |
| Cohort 1 | KDM age accelerated | Binary | Adjusted for Age + Gender | HT vs healthy control | OR | 2.51 (1.58 to 3.99) | <0.001 |

**(added) Supplementary Material 11. The regression analysis of the association between Hashimoto’s thyroiditis and no crp KDMAge and PhenoAge in NHANES**


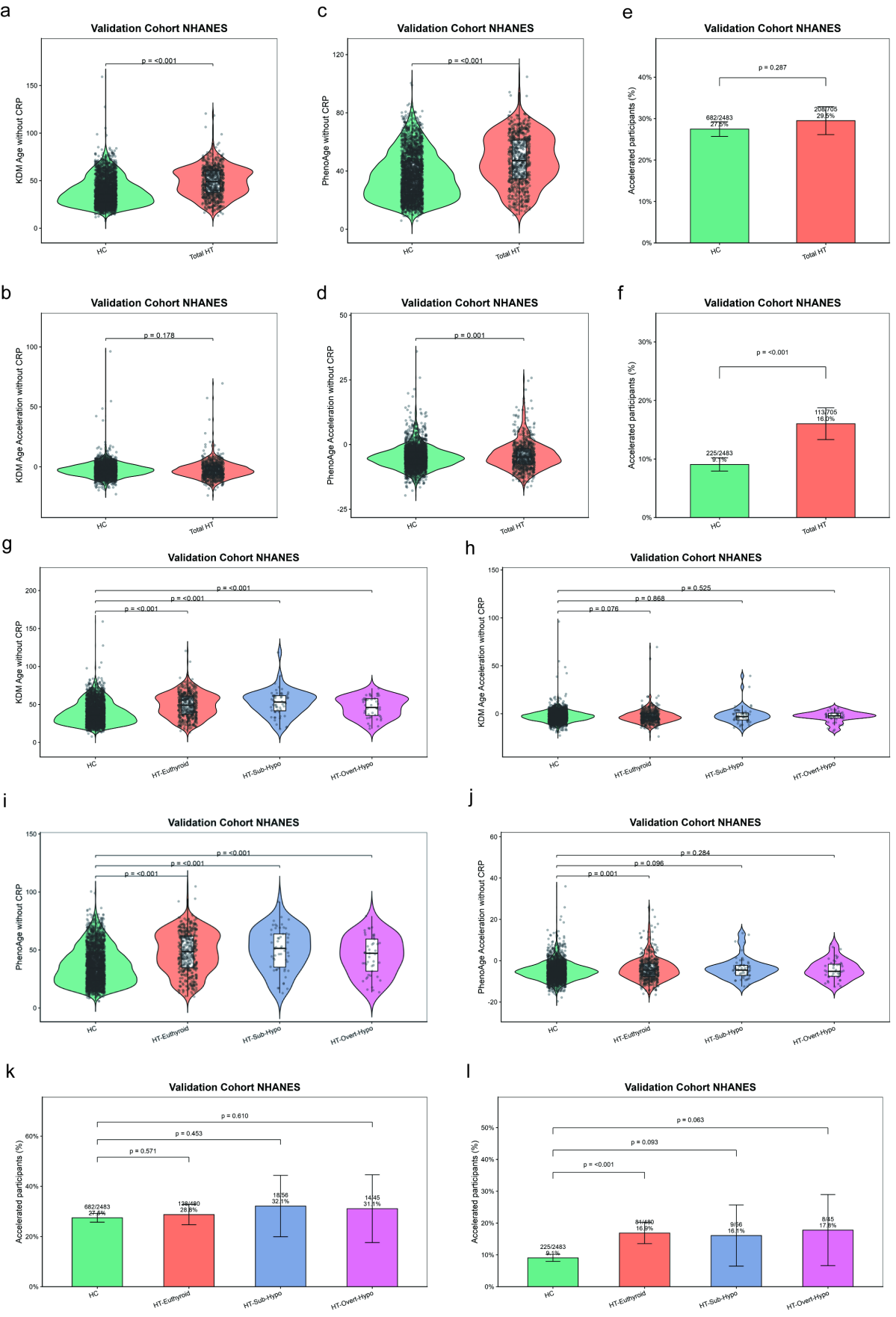


**(added) Supplementary Material 12. The regression analysis of the association between Hashimoto’s thyroiditis and no crp KDMAge and PhenoAge in NHANES**

| Comparison | Subgroup | Model | Outcome | term | estimate | std.error | statistic | p.value | conf.low | conf.high | n | note |
| --- | --- | --- | --- | --- | --- | --- | --- | --- | --- | --- | --- | --- |
| HC vs Total HT | Overall | Model 1: Age + Gender | KDMAge | Group_totalTotal HT | 0.992764743 | 0.258624578 | 3.838632626 | 0.000126127 | 0.485677122 | 1.499852365 | 3188 | Formula: KDMAge ~ Group_total + Age + Gender |
| HC vs Total HT | Overall | Model 1: Age + Gender | PhenoAge | Group_totalTotal HT | 1.143914061 | 0.202154411 | 5.658615402 | 1.66122E-08 | 0.747548024 | 1.540280099 | 3188 | Formula: PhenoAge ~ Group_total + Age + Gender |
| HC vs Total HT | Overall | Model 1: Age + Gender | KDMAge_acc | Group_totalTotal HT | 0.992764743 | 0.258624578 | 3.838632626 | 0.000126127 | 0.485677122 | 1.499852365 | 3188 | Formula: KDMAge_acc ~ Group_total + Age + Gender |
| HC vs Total HT | Overall | Model 1: Age + Gender | PhenoAge_acc | Group_totalTotal HT | 1.143914061 | 0.202154411 | 5.658615402 | 1.66122E-08 | 0.747548024 | 1.540280099 | 3188 | Formula: PhenoAge_acc ~ Group_total + Age + Gender |
| HC vs Total HT | Overall | Model 2: Age + Gender + socioeconomic/lifestyle | KDMAge | Group_totalTotal HT | 1.080252675 | 0.257753934 | 4.191023033 | 2.85288E-05 | 0.574871224 | 1.585634127 | 3188 | Formula: KDMAge ~ Group_total + Age + Gender + Race + PIR_level + Education_level + drinking + Smoking_status + PA |
| HC vs Total HT | Overall | Model 2: Age + Gender + socioeconomic/lifestyle | PhenoAge | Group_totalTotal HT | 1.29513894 | 0.196468228 | 6.592103724 | 5.06022E-11 | 0.909921159 | 1.68035672 | 3188 | Formula: PhenoAge ~ Group_total + Age + Gender + Race + PIR_level + Education_level + drinking + Smoking_status + PA |
| HC vs Total HT | Overall | Model 2: Age + Gender + socioeconomic/lifestyle | KDMAge_acc | Group_totalTotal HT | 1.080252675 | 0.257753934 | 4.191023033 | 2.85288E-05 | 0.574871224 | 1.585634127 | 3188 | Formula: KDMAge_acc ~ Group_total + Age + Gender + Race + PIR_level + Education_level + drinking + Smoking_status + PA |
| HC vs Total HT | Overall | Model 2: Age + Gender + socioeconomic/lifestyle | PhenoAge_acc | Group_totalTotal HT | 1.295138939 | 0.196468228 | 6.592103723 | 5.06022E-11 | 0.909921159 | 1.68035672 | 3188 | Formula: PhenoAge_acc ~ Group_total + Age + Gender + Race + PIR_level + Education_level + drinking + Smoking_status + PA |
| HC vs HT stages | Overall | Model 1: Age + Gender | KDMAge | HT_stageHT-Euthyroid | 0.904908489 | 0.299239889 | 3.024023609 | 0.002515094 | 0.318176856 | 1.491640122 | 3064 | Formula: KDMAge ~ HT_stage + Age + Gender |
| HC vs HT stages | Overall | Model 1: Age + Gender | KDMAge | HT_stageHT-Sub-Hypo | 2.183013042 | 0.787383142 | 2.772491467 | 0.005596598 | 0.639159384 | 3.726866699 | 3064 | Formula: KDMAge ~ HT_stage + Age + Gender |
| HC vs HT stages | Overall | Model 1: Age + Gender | KDMAge | HT_stageHT-Overt-Hypo | 0.521600551 | 0.87540734 | 0.595837535 | 0.551327862 | -1.194845677 | 2.23804678 | 3064 | Formula: KDMAge ~ HT_stage + Age + Gender |
| HC vs HT stages | Overall | Model 1: Age + Gender | PhenoAge | HT_stageHT-Euthyroid | 1.253629185 | 0.234026654 | 5.356779499 | 9.09979E-08 | 0.794763752 | 1.712494618 | 3064 | Formula: PhenoAge ~ HT_stage + Age + Gender |
| HC vs HT stages | Overall | Model 1: Age + Gender | PhenoAge | HT_stageHT-Sub-Hypo | 1.412715504 | 0.615789035 | 2.294155017 | 0.021849077 | 0.205313283 | 2.620117725 | 3064 | Formula: PhenoAge ~ HT_stage + Age + Gender |
| HC vs HT stages | Overall | Model 1: Age + Gender | PhenoAge | HT_stageHT-Overt-Hypo | 1.29594184 | 0.684630154 | 1.892907919 | 0.058464458 | -0.046439919 | 2.638323599 | 3064 | Formula: PhenoAge ~ HT_stage + Age + Gender |
| HC vs HT stages | Overall | Model 1: Age + Gender | KDMAge_acc | HT_stageHT-Euthyroid | 0.904908489 | 0.299239889 | 3.024023609 | 0.002515094 | 0.318176855 | 1.491640122 | 3064 | Formula: KDMAge_acc ~ HT_stage + Age + Gender |
| HC vs HT stages | Overall | Model 1: Age + Gender | KDMAge_acc | HT_stageHT-Sub-Hypo | 2.183013042 | 0.787383142 | 2.772491467 | 0.005596598 | 0.639159385 | 3.726866699 | 3064 | Formula: KDMAge_acc ~ HT_stage + Age + Gender |
| HC vs HT stages | Overall | Model 1: Age + Gender | KDMAge_acc | HT_stageHT-Overt-Hypo | 0.521600551 | 0.87540734 | 0.595837534 | 0.551327863 | -1.194845677 | 2.23804678 | 3064 | Formula: KDMAge_acc ~ HT_stage + Age + Gender |
| HC vs HT stages | Overall | Model 1: Age + Gender | PhenoAge_acc | HT_stageHT-Euthyroid | 1.253629185 | 0.234026654 | 5.356779499 | 9.09979E-08 | 0.794763752 | 1.712494618 | 3064 | Formula: PhenoAge_acc ~ HT_stage + Age + Gender |
| HC vs HT stages | Overall | Model 1: Age + Gender | PhenoAge_acc | HT_stageHT-Sub-Hypo | 1.412715504 | 0.615789035 | 2.294155017 | 0.021849077 | 0.205313283 | 2.620117725 | 3064 | Formula: PhenoAge_acc ~ HT_stage + Age + Gender |
| HC vs HT stages | Overall | Model 1: Age + Gender | PhenoAge_acc | HT_stageHT-Overt-Hypo | 1.29594184 | 0.684630154 | 1.892907919 | 0.058464458 | -0.046439919 | 2.638323599 | 3064 | Formula: PhenoAge_acc ~ HT_stage + Age + Gender |
| HC vs HT stages | Overall | Model 2: Age + Gender + socioeconomic/lifestyle | KDMAge | HT_stageHT-Euthyroid | 0.940346936 | 0.298497622 | 3.150266089 | 0.001647049 | 0.355069553 | 1.525624318 | 3064 | Formula: KDMAge ~ HT_stage + Age + Gender + Race + PIR_level + Education_level + drinking + Smoking_status + PA |
| HC vs HT stages | Overall | Model 2: Age + Gender + socioeconomic/lifestyle | KDMAge | HT_stageHT-Sub-Hypo | 2.415259494 | 0.782160503 | 3.08793334 | 0.002033709 | 0.88164308 | 3.948875907 | 3064 | Formula: KDMAge ~ HT_stage + Age + Gender + Race + PIR_level + Education_level + drinking + Smoking_status + PA |
| HC vs HT stages | Overall | Model 2: Age + Gender + socioeconomic/lifestyle | KDMAge | HT_stageHT-Overt-Hypo | 0.602934688 | 0.870325732 | 0.692769001 | 0.488507394 | -1.103551159 | 2.309420534 | 3064 | Formula: KDMAge ~ HT_stage + Age + Gender + Race + PIR_level + Education_level + drinking + Smoking_status + PA |
| HC vs HT stages | Overall | Model 2: Age + Gender + socioeconomic/lifestyle | PhenoAge | HT_stageHT-Euthyroid | 1.37971951 | 0.227702926 | 6.059296345 | 1.53502E-09 | 0.933252392 | 1.826186627 | 3064 | Formula: PhenoAge ~ HT_stage + Age + Gender + Race + PIR_level + Education_level + drinking + Smoking_status + PA |
| HC vs HT stages | Overall | Model 2: Age + Gender + socioeconomic/lifestyle | PhenoAge | HT_stageHT-Sub-Hypo | 1.634562909 | 0.596655459 | 2.739542369 | 0.006188355 | 0.464674374 | 2.804451443 | 3064 | Formula: PhenoAge ~ HT_stage + Age + Gender + Race + PIR_level + Education_level + drinking + Smoking_status + PA |
| HC vs HT stages | Overall | Model 2: Age + Gender + socioeconomic/lifestyle | PhenoAge | HT_stageHT-Overt-Hypo | 1.34314039 | 0.663910536 | 2.02307437 | 0.043152753 | 0.041381874 | 2.644898906 | 3064 | Formula: PhenoAge ~ HT_stage + Age + Gender + Race + PIR_level + Education_level + drinking + Smoking_status + PA |
| HC vs HT stages | Overall | Model 2: Age + Gender + socioeconomic/lifestyle | KDMAge_acc | HT_stageHT-Euthyroid | 0.940346935 | 0.298497622 | 3.150266088 | 0.001647049 | 0.355069552 | 1.525624318 | 3064 | Formula: KDMAge_acc ~ HT_stage + Age + Gender + Race + PIR_level + Education_level + drinking + Smoking_status + PA |
| HC vs HT stages | Overall | Model 2: Age + Gender + socioeconomic/lifestyle | KDMAge_acc | HT_stageHT-Sub-Hypo | 2.415259494 | 0.782160503 | 3.08793334 | 0.002033709 | 0.881643081 | 3.948875907 | 3064 | Formula: KDMAge_acc ~ HT_stage + Age + Gender + Race + PIR_level + Education_level + drinking + Smoking_status + PA |
| HC vs HT stages | Overall | Model 2: Age + Gender + socioeconomic/lifestyle | KDMAge_acc | HT_stageHT-Overt-Hypo | 0.602934688 | 0.870325732 | 0.692769001 | 0.488507394 | -1.103551159 | 2.309420534 | 3064 | Formula: KDMAge_acc ~ HT_stage + Age + Gender + Race + PIR_level + Education_level + drinking + Smoking_status + PA |
| HC vs HT stages | Overall | Model 2: Age + Gender + socioeconomic/lifestyle | PhenoAge_acc | HT_stageHT-Euthyroid | 1.37971951 | 0.227702926 | 6.059296345 | 1.53502E-09 | 0.933252392 | 1.826186627 | 3064 | Formula: PhenoAge_acc ~ HT_stage + Age + Gender + Race + PIR_level + Education_level + drinking + Smoking_status + PA |
| HC vs HT stages | Overall | Model 2: Age + Gender + socioeconomic/lifestyle | PhenoAge_acc | HT_stageHT-Sub-Hypo | 1.634562909 | 0.596655459 | 2.739542369 | 0.006188355 | 0.464674374 | 2.804451443 | 3064 | Formula: PhenoAge_acc ~ HT_stage + Age + Gender + Race + PIR_level + Education_level + drinking + Smoking_status + PA |
| HC vs HT stages | Overall | Model 2: Age + Gender + socioeconomic/lifestyle | PhenoAge_acc | HT_stageHT-Overt-Hypo | 1.34314039 | 0.663910536 | 2.02307437 | 0.043152753 | 0.041381874 | 2.644898906 | 3064 | Formula: PhenoAge_acc ~ HT_stage + Age + Gender + Race + PIR_level + Education_level + drinking + Smoking_status + PA |
| HC vs Total HT | Male | Model 1: Age + Gender | KDMAge | Group_totalTotal HT | 2.342587017 | 0.396529996 | 5.907717053 | 4.26631E-09 | 1.564788011 | 3.120386023 | 1535 | Formula: KDMAge ~ Group_total + Age |
| HC vs Total HT | Male | Model 1: Age + Gender | PhenoAge | Group_totalTotal HT | 1.976972222 | 0.288040073 | 6.863531875 | 9.72453E-12 | 1.411977682 | 2.541966761 | 1535 | Formula: PhenoAge ~ Group_total + Age |
| HC vs Total HT | Male | Model 1: Age + Gender | KDMAge_acc | Group_totalTotal HT | 2.342587017 | 0.396529996 | 5.907717053 | 4.26631E-09 | 1.564788011 | 3.120386023 | 1535 | Formula: KDMAge_acc ~ Group_total + Age |
| HC vs Total HT | Male | Model 1: Age + Gender | PhenoAge_acc | Group_totalTotal HT | 1.976972222 | 0.288040073 | 6.863531875 | 9.72453E-12 | 1.411977682 | 2.541966761 | 1535 | Formula: PhenoAge_acc ~ Group_total + Age |
| HC vs Total HT | Male | Model 2: Age + Gender + socioeconomic/lifestyle | KDMAge | Group_totalTotal HT | 2.356050969 | 0.394053269 | 5.979016424 | 2.79414E-09 | 1.583104053 | 3.128997886 | 1535 | Formula: KDMAge ~ Group_total + Age + Race + PIR_level + Education_level + drinking + Smoking_status + PA |
| HC vs Total HT | Male | Model 2: Age + Gender + socioeconomic/lifestyle | PhenoAge | Group_totalTotal HT | 2.090270971 | 0.281207864 | 7.433188185 | 1.76354E-13 | 1.53867359 | 2.641868352 | 1535 | Formula: PhenoAge ~ Group_total + Age + Race + PIR_level + Education_level + drinking + Smoking_status + PA |
| HC vs Total HT | Male | Model 2: Age + Gender + socioeconomic/lifestyle | KDMAge_acc | Group_totalTotal HT | 2.356050969 | 0.394053269 | 5.979016424 | 2.79414E-09 | 1.583104053 | 3.128997886 | 1535 | Formula: KDMAge_acc ~ Group_total + Age + Race + PIR_level + Education_level + drinking + Smoking_status + PA |
| HC vs Total HT | Male | Model 2: Age + Gender + socioeconomic/lifestyle | PhenoAge_acc | Group_totalTotal HT | 2.090270971 | 0.281207864 | 7.433188185 | 1.76354E-13 | 1.53867359 | 2.641868352 | 1535 | Formula: PhenoAge_acc ~ Group_total + Age + Race + PIR_level + Education_level + drinking + Smoking_status + PA |
| HC vs HT stages | Male | Model 1: Age + Gender | KDMAge | HT_stageHT-Euthyroid | 1.92112172 | 0.454898797 | 4.223184874 | 2.55428E-05 | 1.028813082 | 2.813430358 | 1498 | Formula: KDMAge ~ HT_stage + Age |
| HC vs HT stages | Male | Model 1: Age + Gender | KDMAge | HT_stageHT-Sub-Hypo | 4.411907692 | 1.114421122 | 3.958923252 | 7.88294E-05 | 2.225910281 | 6.597905103 | 1498 | Formula: KDMAge ~ HT_stage + Age |
| HC vs HT stages | Male | Model 1: Age + Gender | KDMAge | HT_stageHT-Overt-Hypo | 1.112293278 | 1.838730548 | 0.604924566 | 0.545321046 | -2.494476315 | 4.719062871 | 1498 | Formula: KDMAge ~ HT_stage + Age |
| HC vs HT stages | Male | Model 1: Age + Gender | PhenoAge | HT_stageHT-Euthyroid | 2.113040394 | 0.332274562 | 6.35932038 | 2.6873E-10 | 1.461265838 | 2.764814951 | 1498 | Formula: PhenoAge ~ HT_stage + Age |
| HC vs HT stages | Male | Model 1: Age + Gender | PhenoAge | HT_stageHT-Sub-Hypo | 1.743139191 | 0.814013562 | 2.141412961 | 0.032402218 | 0.146407486 | 3.339870896 | 1498 | Formula: PhenoAge ~ HT_stage + Age |
| HC vs HT stages | Male | Model 1: Age + Gender | PhenoAge | HT_stageHT-Overt-Hypo | 1.203136887 | 1.343075407 | 0.895807399 | 0.370500042 | -1.431378293 | 3.837652067 | 1498 | Formula: PhenoAge ~ HT_stage + Age |
| HC vs HT stages | Male | Model 1: Age + Gender | KDMAge_acc | HT_stageHT-Euthyroid | 1.92112172 | 0.454898797 | 4.223184874 | 2.55428E-05 | 1.028813082 | 2.813430358 | 1498 | Formula: KDMAge_acc ~ HT_stage + Age |
| HC vs HT stages | Male | Model 1: Age + Gender | KDMAge_acc | HT_stageHT-Sub-Hypo | 4.411907692 | 1.114421122 | 3.958923252 | 7.88294E-05 | 2.225910281 | 6.597905103 | 1498 | Formula: KDMAge_acc ~ HT_stage + Age |
| HC vs HT stages | Male | Model 1: Age + Gender | KDMAge_acc | HT_stageHT-Overt-Hypo | 1.112293278 | 1.838730548 | 0.604924566 | 0.545321046 | -2.494476315 | 4.719062871 | 1498 | Formula: KDMAge_acc ~ HT_stage + Age |
| HC vs HT stages | Male | Model 1: Age + Gender | PhenoAge_acc | HT_stageHT-Euthyroid | 2.113040394 | 0.332274562 | 6.35932038 | 2.6873E-10 | 1.461265838 | 2.764814951 | 1498 | Formula: PhenoAge_acc ~ HT_stage + Age |
| HC vs HT stages | Male | Model 1: Age + Gender | PhenoAge_acc | HT_stageHT-Sub-Hypo | 1.743139191 | 0.814013562 | 2.141412961 | 0.032402218 | 0.146407486 | 3.339870896 | 1498 | Formula: PhenoAge_acc ~ HT_stage + Age |
| HC vs HT stages | Male | Model 1: Age + Gender | PhenoAge_acc | HT_stageHT-Overt-Hypo | 1.203136887 | 1.343075407 | 0.895807399 | 0.370500042 | -1.431378293 | 3.837652067 | 1498 | Formula: PhenoAge_acc ~ HT_stage + Age |
| HC vs HT stages | Male | Model 2: Age + Gender + socioeconomic/lifestyle | KDMAge | HT_stageHT-Euthyroid | 1.809278764 | 0.452850905 | 3.995307823 | 6.77906E-05 | 0.920979864 | 2.697577665 | 1498 | Formula: KDMAge ~ HT_stage + Age + Race + PIR_level + Education_level + drinking + Smoking_status + PA |
| HC vs HT stages | Male | Model 2: Age + Gender + socioeconomic/lifestyle | KDMAge | HT_stageHT-Sub-Hypo | 4.854565831 | 1.105293769 | 4.392104585 | 1.2025E-05 | 2.686454365 | 7.022677296 | 1498 | Formula: KDMAge ~ HT_stage + Age + Race + PIR_level + Education_level + drinking + Smoking_status + PA |
| HC vs HT stages | Male | Model 2: Age + Gender + socioeconomic/lifestyle | KDMAge | HT_stageHT-Overt-Hypo | 1.516905578 | 1.822515648 | 0.832314159 | 0.405366101 | -2.058087047 | 5.091898202 | 1498 | Formula: KDMAge ~ HT_stage + Age + Race + PIR_level + Education_level + drinking + Smoking_status + PA |
| HC vs HT stages | Male | Model 2: Age + Gender + socioeconomic/lifestyle | PhenoAge | HT_stageHT-Euthyroid | 2.134555038 | 0.325224998 | 6.563317858 | 7.26238E-11 | 1.49660333 | 2.772506746 | 1498 | Formula: PhenoAge ~ HT_stage + Age + Race + PIR_level + Education_level + drinking + Smoking_status + PA |
| HC vs HT stages | Male | Model 2: Age + Gender + socioeconomic/lifestyle | PhenoAge | HT_stageHT-Sub-Hypo | 2.033677356 | 0.793791422 | 2.561979508 | 0.010506157 | 0.476599653 | 3.590755059 | 1498 | Formula: PhenoAge ~ HT_stage + Age + Race + PIR_level + Education_level + drinking + Smoking_status + PA |
| HC vs HT stages | Male | Model 2: Age + Gender + socioeconomic/lifestyle | PhenoAge | HT_stageHT-Overt-Hypo | 1.633530121 | 1.308880343 | 1.248036255 | 0.212215458 | -0.933930727 | 4.20099097 | 1498 | Formula: PhenoAge ~ HT_stage + Age + Race + PIR_level + Education_level + drinking + Smoking_status + PA |
| HC vs HT stages | Male | Model 2: Age + Gender + socioeconomic/lifestyle | KDMAge_acc | HT_stageHT-Euthyroid | 1.809278764 | 0.452850905 | 3.995307822 | 6.77906E-05 | 0.920979863 | 2.697577665 | 1498 | Formula: KDMAge_acc ~ HT_stage + Age + Race + PIR_level + Education_level + drinking + Smoking_status + PA |
| HC vs HT stages | Male | Model 2: Age + Gender + socioeconomic/lifestyle | KDMAge_acc | HT_stageHT-Sub-Hypo | 4.854565831 | 1.105293769 | 4.392104585 | 1.2025E-05 | 2.686454365 | 7.022677297 | 1498 | Formula: KDMAge_acc ~ HT_stage + Age + Race + PIR_level + Education_level + drinking + Smoking_status + PA |
| HC vs HT stages | Male | Model 2: Age + Gender + socioeconomic/lifestyle | KDMAge_acc | HT_stageHT-Overt-Hypo | 1.516905578 | 1.822515648 | 0.832314159 | 0.405366101 | -2.058087047 | 5.091898202 | 1498 | Formula: KDMAge_acc ~ HT_stage + Age + Race + PIR_level + Education_level + drinking + Smoking_status + PA |
| HC vs HT stages | Male | Model 2: Age + Gender + socioeconomic/lifestyle | PhenoAge_acc | HT_stageHT-Euthyroid | 2.134555038 | 0.325224998 | 6.563317859 | 7.26238E-11 | 1.496603331 | 2.772506746 | 1498 | Formula: PhenoAge_acc ~ HT_stage + Age + Race + PIR_level + Education_level + drinking + Smoking_status + PA |
| HC vs HT stages | Male | Model 2: Age + Gender + socioeconomic/lifestyle | PhenoAge_acc | HT_stageHT-Sub-Hypo | 2.033677356 | 0.793791422 | 2.561979508 | 0.010506157 | 0.476599653 | 3.590755059 | 1498 | Formula: PhenoAge_acc ~ HT_stage + Age + Race + PIR_level + Education_level + drinking + Smoking_status + PA |
| HC vs HT stages | Male | Model 2: Age + Gender + socioeconomic/lifestyle | PhenoAge_acc | HT_stageHT-Overt-Hypo | 1.633530121 | 1.308880343 | 1.248036255 | 0.212215458 | -0.933930727 | 4.20099097 | 1498 | Formula: PhenoAge_acc ~ HT_stage + Age + Race + PIR_level + Education_level + drinking + Smoking_status + PA |
| HC vs Total HT | Female | Model 1: Age + Gender | KDMAge | Group_totalTotal HT | 0.251150953 | 0.341657802 | 0.735095033 | 0.462386085 | -0.418977604 | 0.92127951 | 1653 | Formula: KDMAge ~ Group_total + Age |
| HC vs Total HT | Female | Model 1: Age + Gender | PhenoAge | Group_totalTotal HT | 0.700414795 | 0.281640241 | 2.486913065 | 0.012983884 | 0.148004848 | 1.252824742 | 1653 | Formula: PhenoAge ~ Group_total + Age |
| HC vs Total HT | Female | Model 1: Age + Gender | KDMAge_acc | Group_totalTotal HT | 0.251150953 | 0.341657802 | 0.735095032 | 0.462386085 | -0.418977604 | 0.92127951 | 1653 | Formula: KDMAge_acc ~ Group_total + Age |
| HC vs Total HT | Female | Model 1: Age + Gender | PhenoAge_acc | Group_totalTotal HT | 0.700414795 | 0.281640241 | 2.486913065 | 0.012983884 | 0.148004848 | 1.252824742 | 1653 | Formula: PhenoAge_acc ~ Group_total + Age |
| HC vs Total HT | Female | Model 2: Age + Gender + socioeconomic/lifestyle | KDMAge | Group_totalTotal HT | 0.338756491 | 0.342863624 | 0.988021087 | 0.32328851 | -0.333741698 | 1.01125468 | 1653 | Formula: KDMAge ~ Group_total + Age + Race + PIR_level + Education_level + drinking + Smoking_status + PA |
| HC vs Total HT | Female | Model 2: Age + Gender + socioeconomic/lifestyle | PhenoAge | Group_totalTotal HT | 0.900853489 | 0.273251381 | 3.296793906 | 0.000998917 | 0.364893865 | 1.436813113 | 1653 | Formula: PhenoAge ~ Group_total + Age + Race + PIR_level + Education_level + drinking + Smoking_status + PA |
| HC vs Total HT | Female | Model 2: Age + Gender + socioeconomic/lifestyle | KDMAge_acc | Group_totalTotal HT | 0.338756491 | 0.342863624 | 0.988021087 | 0.32328851 | -0.333741698 | 1.01125468 | 1653 | Formula: KDMAge_acc ~ Group_total + Age + Race + PIR_level + Education_level + drinking + Smoking_status + PA |
| HC vs Total HT | Female | Model 2: Age + Gender + socioeconomic/lifestyle | PhenoAge_acc | Group_totalTotal HT | 0.900853489 | 0.273251381 | 3.296793906 | 0.000998917 | 0.364893865 | 1.436813113 | 1653 | Formula: PhenoAge_acc ~ Group_total + Age + Race + PIR_level + Education_level + drinking + Smoking_status + PA |
| HC vs HT stages | Female | Model 1: Age + Gender | KDMAge | HT_stageHT-Euthyroid | 0.362185238 | 0.399655642 | 0.906243276 | 0.364946963 | -0.421733251 | 1.146103727 | 1566 | Formula: KDMAge ~ HT_stage + Age |
| HC vs HT stages | Female | Model 1: Age + Gender | KDMAge | HT_stageHT-Sub-Hypo | 0.388639483 | 1.102696732 | 0.352444577 | 0.724552454 | -1.774283457 | 2.551562424 | 1566 | Formula: KDMAge ~ HT_stage + Age |
| HC vs HT stages | Female | Model 1: Age + Gender | KDMAge | HT_stageHT-Overt-Hypo | 0.331760711 | 1.021252538 | 0.324856682 | 0.745333072 | -1.671410676 | 2.334932099 | 1566 | Formula: KDMAge ~ HT_stage + Age |
| HC vs HT stages | Female | Model 1: Age + Gender | PhenoAge | HT_stageHT-Euthyroid | 0.802505189 | 0.328598628 | 2.442204929 | 0.01470816 | 0.157963956 | 1.447046421 | 1566 | Formula: PhenoAge ~ HT_stage + Age |
| HC vs HT stages | Female | Model 1: Age + Gender | PhenoAge | HT_stageHT-Sub-Hypo | 1.153391964 | 0.906642107 | 1.272157951 | 0.203506479 | -0.624972798 | 2.931756726 | 1566 | Formula: PhenoAge ~ HT_stage + Age |
| HC vs HT stages | Female | Model 1: Age + Gender | PhenoAge | HT_stageHT-Overt-Hypo | 1.301416343 | 0.839678332 | 1.549898686 | 0.12136854 | -0.345599989 | 2.948432675 | 1566 | Formula: PhenoAge ~ HT_stage + Age |
| HC vs HT stages | Female | Model 1: Age + Gender | KDMAge_acc | HT_stageHT-Euthyroid | 0.362185238 | 0.399655642 | 0.906243276 | 0.364946963 | -0.421733251 | 1.146103727 | 1566 | Formula: KDMAge_acc ~ HT_stage + Age |
| HC vs HT stages | Female | Model 1: Age + Gender | KDMAge_acc | HT_stageHT-Sub-Hypo | 0.388639484 | 1.102696732 | 0.352444577 | 0.724552454 | -1.774283457 | 2.551562424 | 1566 | Formula: KDMAge_acc ~ HT_stage + Age |
| HC vs HT stages | Female | Model 1: Age + Gender | KDMAge_acc | HT_stageHT-Overt-Hypo | 0.331760711 | 1.021252538 | 0.324856682 | 0.745333073 | -1.671410676 | 2.334932099 | 1566 | Formula: KDMAge_acc ~ HT_stage + Age |
| HC vs HT stages | Female | Model 1: Age + Gender | PhenoAge_acc | HT_stageHT-Euthyroid | 0.802505189 | 0.328598628 | 2.442204929 | 0.01470816 | 0.157963956 | 1.447046421 | 1566 | Formula: PhenoAge_acc ~ HT_stage + Age |
| HC vs HT stages | Female | Model 1: Age + Gender | PhenoAge_acc | HT_stageHT-Sub-Hypo | 1.153391964 | 0.906642107 | 1.272157951 | 0.203506479 | -0.624972798 | 2.931756726 | 1566 | Formula: PhenoAge_acc ~ HT_stage + Age |
| HC vs HT stages | Female | Model 1: Age + Gender | PhenoAge_acc | HT_stageHT-Overt-Hypo | 1.301416343 | 0.839678332 | 1.549898686 | 0.12136854 | -0.345599989 | 2.948432675 | 1566 | Formula: PhenoAge_acc ~ HT_stage + Age |
| HC vs HT stages | Female | Model 2: Age + Gender + socioeconomic/lifestyle | KDMAge | HT_stageHT-Euthyroid | 0.425528628 | 0.40109694 | 1.060912178 | 0.288895521 | -0.361222869 | 1.212280124 | 1566 | Formula: KDMAge ~ HT_stage + Age + Race + PIR_level + Education_level + drinking + Smoking_status + PA |
| HC vs HT stages | Female | Model 2: Age + Gender + socioeconomic/lifestyle | KDMAge | HT_stageHT-Sub-Hypo | 0.366437272 | 1.100984125 | 0.332827026 | 0.739309982 | -1.793142675 | 2.526017219 | 1566 | Formula: KDMAge ~ HT_stage + Age + Race + PIR_level + Education_level + drinking + Smoking_status + PA |
| HC vs HT stages | Female | Model 2: Age + Gender + socioeconomic/lifestyle | KDMAge | HT_stageHT-Overt-Hypo | 0.467821628 | 1.022722553 | 0.457427703 | 0.647427929 | -1.538248275 | 2.473891531 | 1566 | Formula: KDMAge ~ HT_stage + Age + Race + PIR_level + Education_level + drinking + Smoking_status + PA |
| HC vs HT stages | Female | Model 2: Age + Gender + socioeconomic/lifestyle | PhenoAge | HT_stageHT-Euthyroid | 1.041531281 | 0.318759749 | 3.267449187 | 0.001109018 | 0.416284155 | 1.666778408 | 1566 | Formula: PhenoAge ~ HT_stage + Age + Race + PIR_level + Education_level + drinking + Smoking_status + PA |
| HC vs HT stages | Female | Model 2: Age + Gender + socioeconomic/lifestyle | PhenoAge | HT_stageHT-Sub-Hypo | 1.243359389 | 0.874974073 | 1.421024266 | 0.155511368 | -0.472901925 | 2.959620703 | 1566 | Formula: PhenoAge ~ HT_stage + Age + Race + PIR_level + Education_level + drinking + Smoking_status + PA |
| HC vs HT stages | Female | Model 2: Age + Gender + socioeconomic/lifestyle | PhenoAge | HT_stageHT-Overt-Hypo | 1.251674171 | 0.812778038 | 1.539995068 | 0.123766196 | -0.342589646 | 2.845937987 | 1566 | Formula: PhenoAge ~ HT_stage + Age + Race + PIR_level + Education_level + drinking + Smoking_status + PA |
| HC vs HT stages | Female | Model 2: Age + Gender + socioeconomic/lifestyle | KDMAge_acc | HT_stageHT-Euthyroid | 0.425528628 | 0.40109694 | 1.060912177 | 0.288895521 | -0.361222869 | 1.212280124 | 1566 | Formula: KDMAge_acc ~ HT_stage + Age + Race + PIR_level + Education_level + drinking + Smoking_status + PA |
| HC vs HT stages | Female | Model 2: Age + Gender + socioeconomic/lifestyle | KDMAge_acc | HT_stageHT-Sub-Hypo | 0.366437273 | 1.100984125 | 0.332827027 | 0.739309981 | -1.793142674 | 2.52601722 | 1566 | Formula: KDMAge_acc ~ HT_stage + Age + Race + PIR_level + Education_level + drinking + Smoking_status + PA |
| HC vs HT stages | Female | Model 2: Age + Gender + socioeconomic/lifestyle | KDMAge_acc | HT_stageHT-Overt-Hypo | 0.467821628 | 1.022722553 | 0.457427702 | 0.647427929 | -1.538248276 | 2.473891531 | 1566 | Formula: KDMAge_acc ~ HT_stage + Age + Race + PIR_level + Education_level + drinking + Smoking_status + PA |
| HC vs HT stages | Female | Model 2: Age + Gender + socioeconomic/lifestyle | PhenoAge_acc | HT_stageHT-Euthyroid | 1.041531281 | 0.318759749 | 3.267449187 | 0.001109018 | 0.416284154 | 1.666778408 | 1566 | Formula: PhenoAge_acc ~ HT_stage + Age + Race + PIR_level + Education_level + drinking + Smoking_status + PA |
| HC vs HT stages | Female | Model 2: Age + Gender + socioeconomic/lifestyle | PhenoAge_acc | HT_stageHT-Sub-Hypo | 1.243359389 | 0.874974073 | 1.421024266 | 0.155511368 | -0.472901925 | 2.959620703 | 1566 | Formula: PhenoAge_acc ~ HT_stage + Age + Race + PIR_level + Education_level + drinking + Smoking_status + PA |
| HC vs HT stages | Female | Model 2: Age + Gender + socioeconomic/lifestyle | PhenoAge_acc | HT_stageHT-Overt-Hypo | 1.251674171 | 0.812778038 | 1.539995068 | 0.123766196 | -0.342589646 | 2.845937987 | 1566 | Formula: PhenoAge_acc ~ HT_stage + Age + Race + PIR_level + Education_level + drinking + Smoking_status + PA |
