# Supplementary material for: Metabolic dysregulation and biological age acceleration in Hashimoto’s thyroiditis: a cross-sectional study based on clinical biomarker aging indices and metabolomics
Source: Front Endocrinol (Lausanne). 2026 Jun 26;17:1874574. doi: 10.3389/fendo.2026.1874574 (PMC13350340; doi:10.3389/fendo.2026.1874574)
Supplement: Supplementary file 2 [file SupplementaryFile1.docx]

**Figure S1.** Sex-stratified comparisons of biological age, age acceleration, and accelerated proportions between patients with HT and healthy controls in discovery cohorts 1 and 2.
(a-c) Distributions of biological age and chronological age: KDM age in discovery cohort 1 (a), PhenoAge in discovery cohort 1 (b), and KDM age in discovery cohort 2 (c).
(d-h) Sex-stratified comparisons between healthy controls and HT patients in discovery cohort 1 for KDM biological age (d), KDM age acceleration (e), PhenoAge (f), PhenoAge acceleration (g), and the proportion with KDM age or PhenoAge acceleration (h).
(i-j) Sex-stratified comparisons between healthy controls and HT patients in discovery cohort 2 for KDM biological age (i) and KDM age acceleration (j).


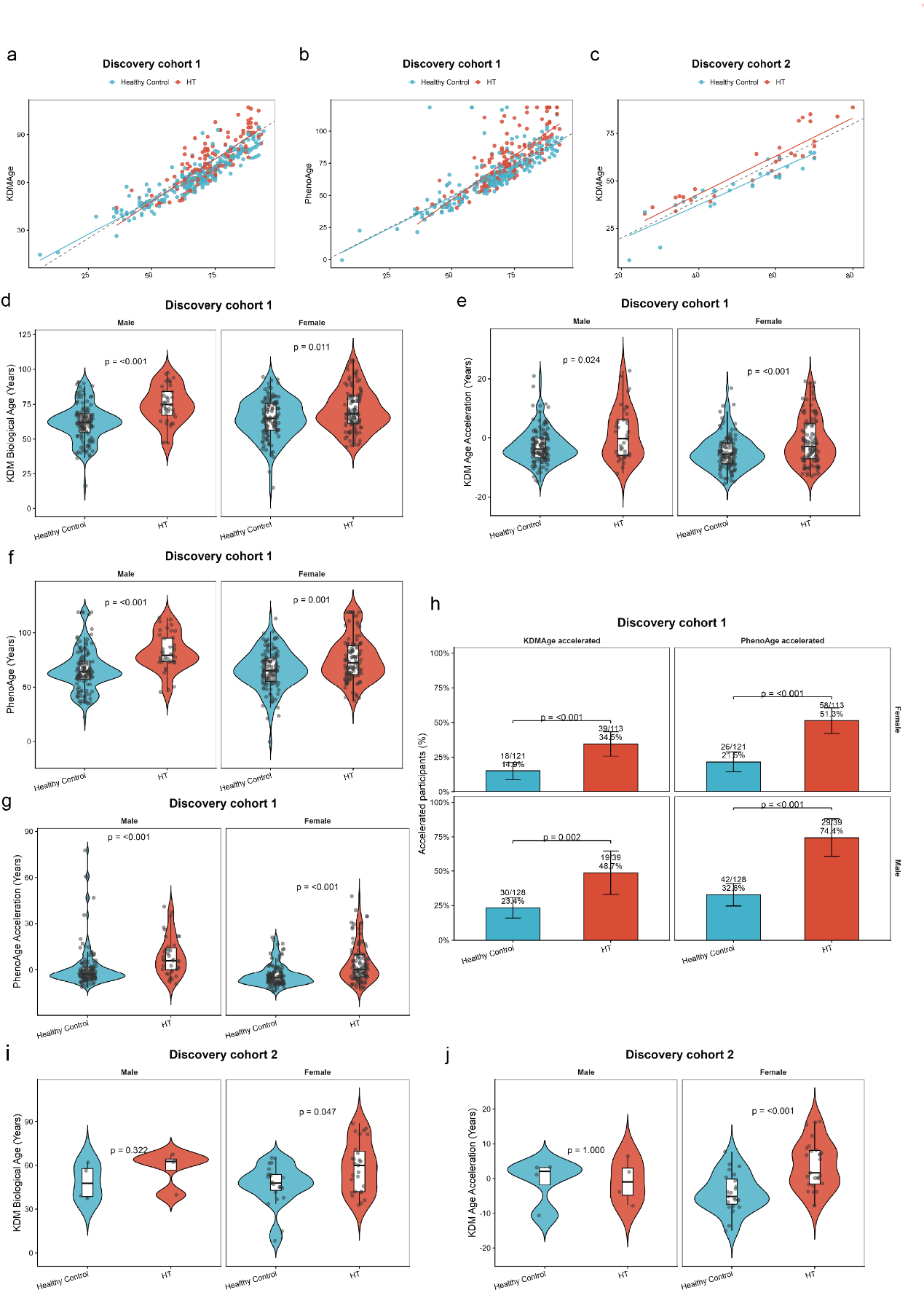


**Figure S2.** Biological age, age acceleration, and accelerated proportions in male HT patients and healthy controls in the NHANES cohort.
(a-f) Comparisons between male healthy controls and male HT patients in NHANES for KDM biological age (a), KDM age acceleration (b), PhenoAge (c), PhenoAge acceleration (d), the proportion with KDM age acceleration (e), and the proportion with PhenoAge acceleration (f).
(g-i) Comparisons among male healthy controls and male HT patients at different stages in NHANES for KDM biological age (g), KDM age acceleration (h), and PhenoAge (i). P <0.05 was considered statistically significant.


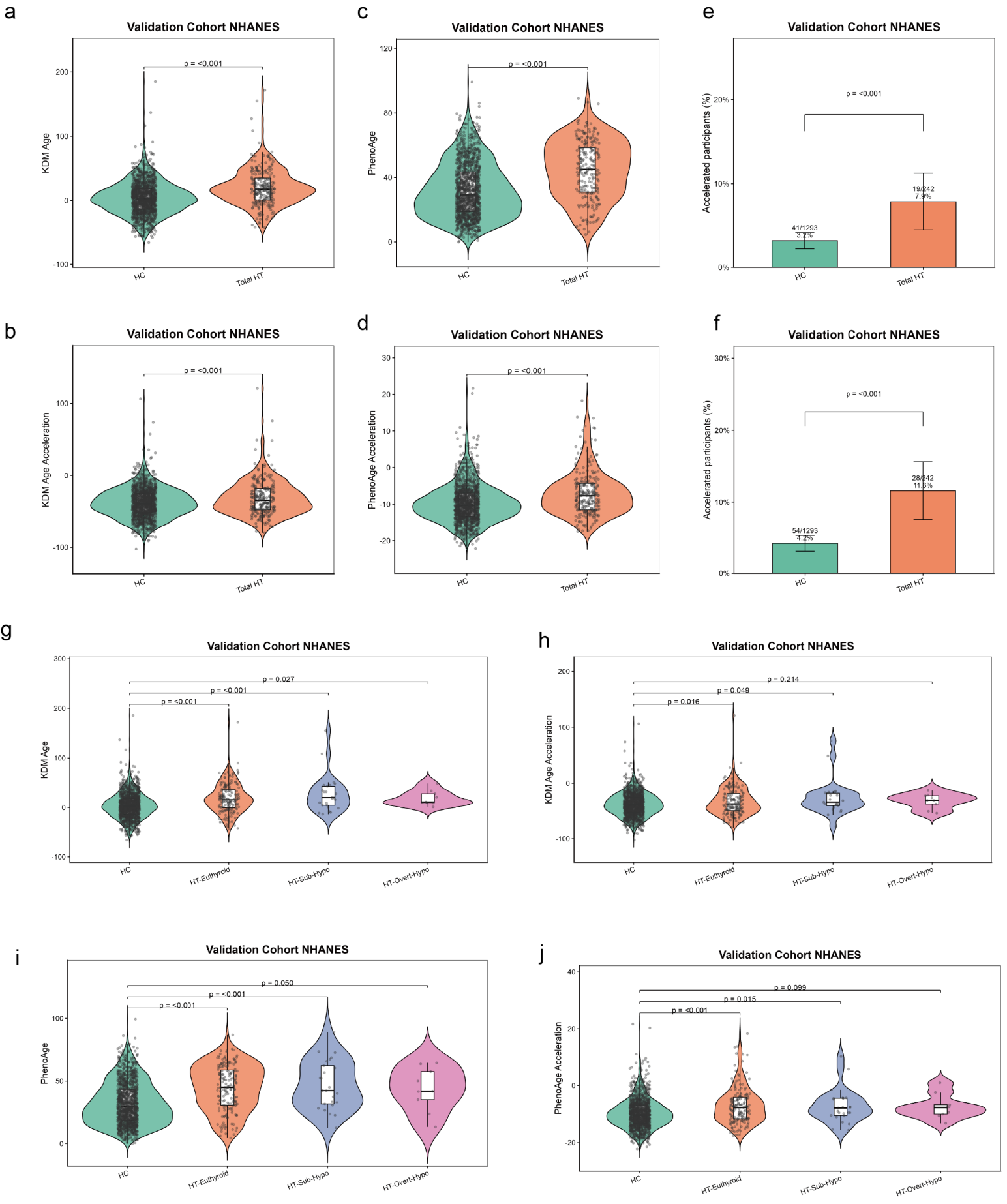
**Figure S3.** Biological age, age acceleration, and accelerated proportions in female HT patients and healthy controls in the NHANES cohort.
(a-f) Comparisons between female healthy controls and female HT patients in NHANES for KDM biological age (a), KDM age acceleration (b), PhenoAge (c), PhenoAge acceleration (d), the proportion with KDM age acceleration (e), and the proportion with PhenoAge acceleration (f).
(g-i) Comparisons among female healthy controls and female HT patients at different stages in NHANES for KDM biological age (g), KDM age acceleration (h), and PhenoAge (i). P <0.05 was considered statistically significant.


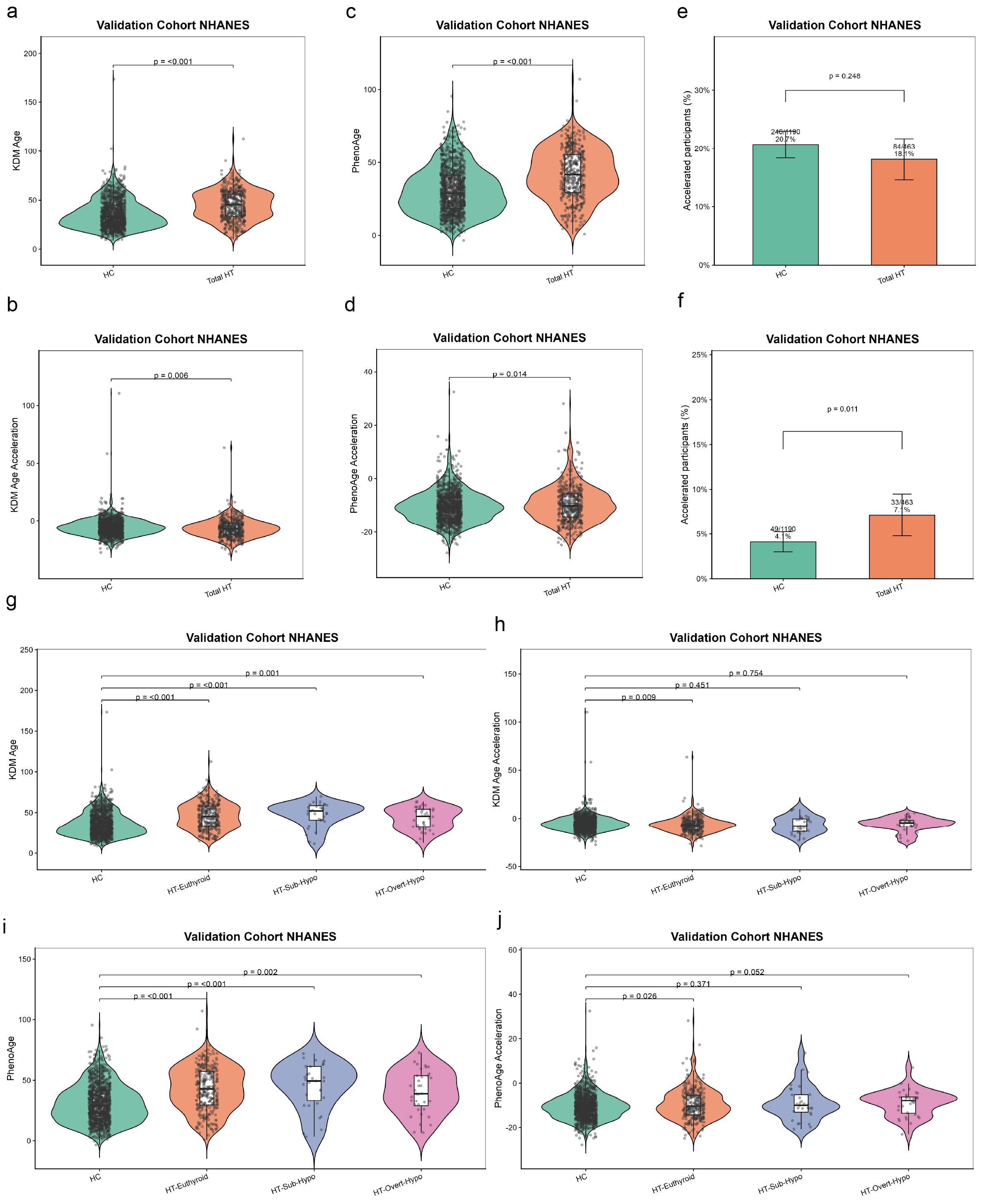


**Figure S4.** Sex-stratified metabolic age features and metabolic age acceleration analyses.
(a) Test-set and training-set performance of the random forest metabolic age model validated by five-fold cross-validation.
(b) Sex-stratified correlations between chronological age and predicted metabolic age. Scatterplots show the relationship between chronological age and random forest-predicted metabolic age in females (left) and males (right). Each point represents one participant, colored by CON, EHT, or DHT. Black lines indicate fitted trends.
(c) Sex-stratified comparisons of metabolic age across groups. Distributions of metabolic age were compared among CON, EHT, and DHT in females (left) and males (right). Each point represents one participant; violin plot width indicates density; overlaid boxplots show medians and interquartile ranges.
(d) Sex-stratified differences in MAA across groups. MAA levels were compared among CON, EHT, and DHT in females (left) and males (right). Gray points indicate individual values, and colored bars with error bars indicate group summary statistics.
(e) Sex-stratified proportions of metabolic age acceleration across groups. Stacked bar charts show accelerated and non-accelerated metabolic age status among CON, EHT, and DHT in females (left) and males (right). Red indicates acceleration and blue indicates non-acceleration.


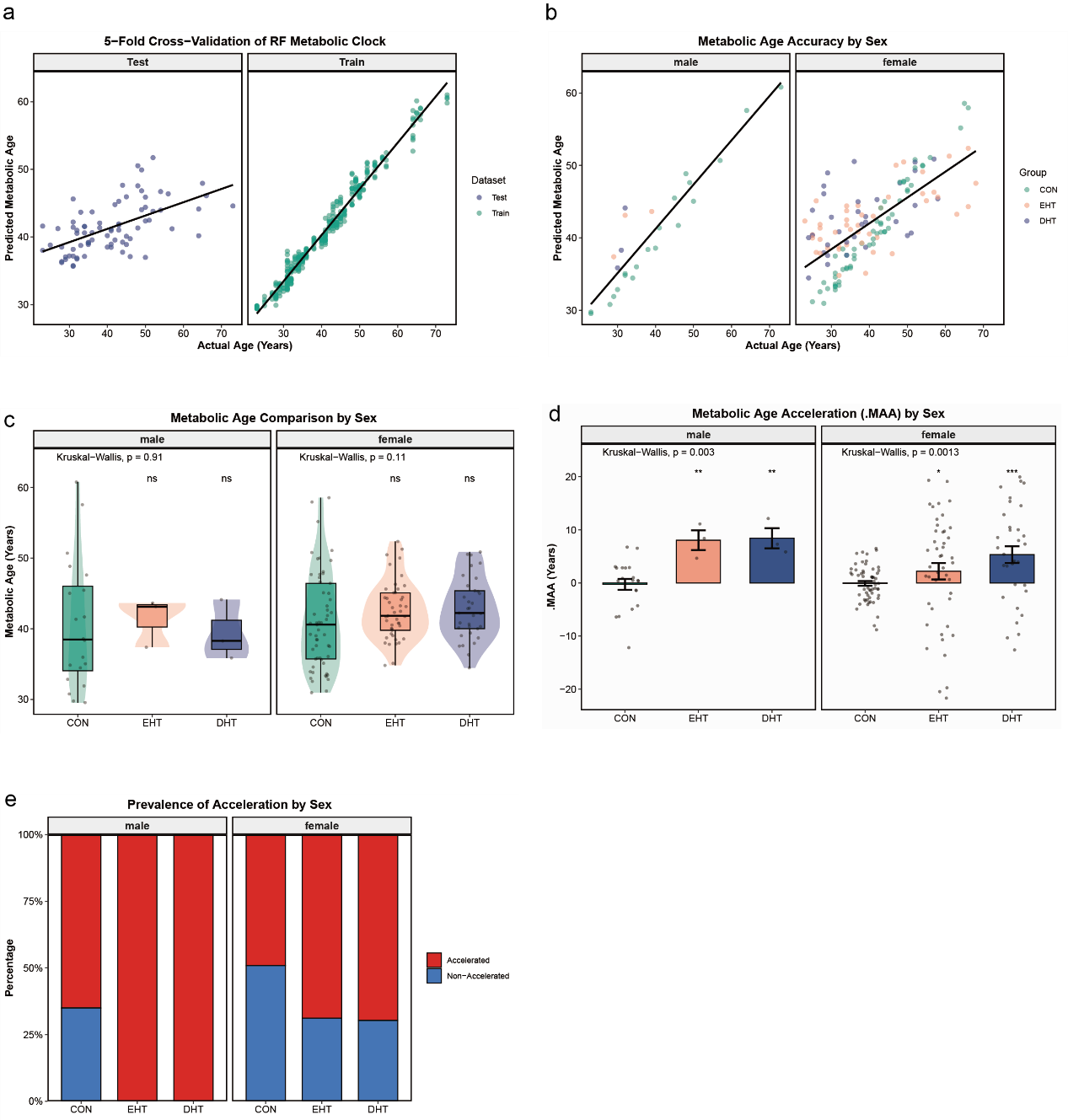


**Figure S5.** PCA and PLS-DA of the HT metabolomics cohort.
The left panel shows principal component analysis (PCA), and the right panel shows partial least squares-discriminant analysis (PLS-DA). Each point represents one participant, with colors indicating CON, EHT, and DHT; dashed ellipses indicate the overall clustering ranges of groups. PCA showed partial overlap among the three groups but suggested distributional differences, whereas PLS-DA further showed partial separation, suggesting metabolomic differences among study groups.
